# Multi-Strategy Improved Whale Optimization Algorithm and Its Engineering Applications

**DOI:** 10.3390/biomimetics10010047

**Published:** 2025-01-13

**Authors:** Yu Zhou, Zijun Hao

**Affiliations:** School of Mathematics and Information Science, North Minzu University, Yinchuan 750021, China; zhouyu20010112@126.com

**Keywords:** improved whale optimization algorithm, dynamic elastic boundary optimization strategy, improved random searching strategy, combined mutation mechanism

## Abstract

The Whale Optimization Algorithm (WOA) is recognized for its simplicity, few control parameters, and effective local optima avoidance. However, it struggles with global search efficiency and slow convergence. This paper introduces the Improved WOA (ImWOA) to overcome these challenges. Initially, ImWOA utilizes a dynamic elastic boundary optimization strategy, which leverages boundary information and the current optimal position to guide solutions that exceed the boundaries back within permissible limits, gradually converging towards the optimal solution. Subsequently, ImWOA integrates an advanced random searching strategy that equilibrates global and local searches by focusing on the current optimal location and the mean position of all individuals. Lastly, a combined mutation mechanism is employed to enhance population diversity, prevent the algorithm from stagnating in local optima, and consequently augment its overall search capability. Performance evaluations on CEC2017 benchmark functions show ImWOA outperforming five metaheuristic algorithms and three WOA variants in optimization accuracy, stability, and convergence speed. ImWOA excelled in 25 out of 29 test functions in 30D and 26 out of 29 in 100D scenarios. Furthermore, its efficacy in addressing complex challenges is corroborated by real-world applications in reducer design, vehicle side impact design, and welded beam design, highlighting its potential utility across various engineering domains.

## 1. Introduction

The core of optimization problems is centered on identifying a solution that meets all or most of the specified constraints while optimizing a particular performance metric within a set of predefined conditions. As advancements in science and technology continue at a rapid pace, the complexity and diversity of the optimization challenges we face have increased significantly. These challenges extend beyond traditional linear or quadratic programming, and encompass features such as nonlinearity, multi-objectivity, dynamic changes, and high uncertainty, often involving high-dimensional spaces.

To effectively address these increasingly complex and critical optimization challenges, researchers and engineers have developed a diverse range of optimization algorithms. Swarm intelligence algorithms, in particular, have gained prominence due to their unique advantages and have demonstrated efficacy in addressing complex optimization problems [1,2]. Prominent examples of swarm intelligence algorithms include Particle Swarm Optimization (PSO) [3], Chameleon Swarm Algorithm (CSA) [4], Bald Eagle Search (BES) [5], Harris Hawks Optimization (HHO) [6], and Elephant Herding Optimization (EHO) [7].

Researchers have identified the Whale Optimization Algorithm (WOA) [8] as particularly appealing due to its straightforward design, robust adaptability, and minimal control parameters. This algorithm has demonstrated efficacy in addressing a diverse array of complex optimization problems, such as engineering design [9,10,11], image segmentation [12,13,14,15], path planning [16], feature selection [17,18] and workshop scheduling [19,20,21], fuzzy programming [22] and DNA fragment assembly [23].

Nonetheless, the limitations of the WOA become apparent when applied to high-dimensional data and complex problems. Specifically, its optimization speed is relatively slow, often failing to meet the demand for rapid solutions. Additionally, the optimization accuracy is insufficiently high, potentially leading to suboptimal solution quality. Furthermore, the WOA exhibits deficiencies in search and exploitation capabilities, hindering its ability to thoroughly and deeply explore the solution space, thereby limiting its effectiveness in practical applications [24,25]. To address these constraints and enhance the performance of the WOA, numerous scholars have conducted comprehensive studies and proposed a variety of effective enhancement techniques. These methods are designed to optimize and enhance the WOA by refining its algorithmic structure, parameter configurations, search strategies, and other relevant aspects to achieve superior solution outcomes.

Lee et al. [26] introduced an innovative hybrid optimization approach named GWOATEO, which synergistically integrates the genetic algorithm with heat exchange optimization techniques. This algorithm utilizes memory-based crossover techniques and position updating methods for its leading solutions, thereby augmenting its search capabilities. Furthermore, the study implemented an antagonistic learning strategy to generate the inverse of the global optimal solution, thus accelerating the algorithm’s performance and further improving its search efficiency. Similarly, Li et al. [27] proposed a multi-leader guided multi-objective WOA that incorporates an opposition-based learning strategy to enhance the initial population distribution. Their research demonstrated the effectiveness of this oppositional learning technique in generating a contrasting solution to the global optimum, thereby improving the overall optimal solution beyond merely optimizing the initial population distribution. Liu et al. [28] advanced global optimization by employing an elite search strategy and implementing an adaptive variable-speed method to achieve a balance between exploration and exploitation within the algorithm. Their research successfully enhanced the equilibrium between algorithmic progression and search performance by incorporating historical individual optimal solutions, adaptive inertia weights, and refined global optima. Ling et al. [29] introduced a WOA that integrates the Lévy flight trajectory mechanism, which facilitates population diversification, reduces premature convergence, and enhances the algorithm’s ability to escape local optima. Xiong et al. [30] developed an improved WOA by combining two prey search methods to achieve a balance between local exploitation and global exploration. Yang et al. [31] refined the WOA by incorporating adaptive nonlinear inertia weights to control convergence speed and implementing a finite mutation mechanism to improve convergence efficiency, thereby effectively addressing economic load scheduling problems. Ning et al. [32] incorporated the Gaussian variational approach and a dynamic penalty function method into the initial population creation and convergence processes. These enhancements significantly improved the algorithm’s global search capability, stability, convergence rate, accuracy, and robustness. Additionally, they exhibited beneficial effects in addressing more complex constrained optimization problems. Jiang et al. [33] utilized scheduling principles, nonlinear convergence elements, and mutation techniques to improve the quality of initial solutions, balance exploration and exploitation, and prevent stagnation. These methodologies have proven effective in addressing the energy-saving scheduling problem.

Despite the performance improvements in the WOA achieved through these variants, there remains potential for further enhancement of its global exploration ability and convergence rate. To tackle these challenges, this study introduces the Improved WOA algorithm, referred to as ImWOA, which integrates multiple strategies. Firstly, a dynamic elastic boundary optimization strategy is employed, setting it apart from traditional boundary management methods. This strategy utilizes information such as the upper limit, lower limit, and the current optimal position to guide individuals who exceed the boundaries back within the permissible range, gradually steering them towards the current optimal solution. This approach mitigates performance degradation when individuals surpass the defined search space and enhances search efficiency by directing them towards the optimal solution. Secondly, an enhanced random search strategy is implemented, which emphasizes both the current optimal position and the mean position of all individuals. This method achieves a balance between global and local searches, thereby maintaining diversity and exploration throughout the entire search domain. Finally, a combined mutation mechanism is introduced to enhance population diversity, reduce the likelihood of convergence to local optima, and thereby improve both the algorithm’s ability to perform global searches and computational efficiency. To evaluate the efficacy of the ImWOA, we conducted comparative experiments using the CEC2017 test functions and three engineering application scenarios. The findings indicate a significant enhancement in the performance of the WOA due to the implementation of these innovative strategies.

The structure of the subsequent sections of this paper is organized as follows: Section 2 provides a concise overview of the WOA. Section 3 presents a comprehensive exposition of the ImWOA methodology. Section 4 describes the comparative experimental analyses. In Section 5, three different engineering design problems are addressed using the ImWOA algorithm, with a detailed analysis of the results. Finally, Section 6 concludes the paper by summarizing the main findings and suggesting potential directions for future research.

## 2. The Original WOA

In 2016, Mirjalili and Lewis [8] introduced the WOA, which was inspired by the predatory strategies of humpback whales. This algorithm integrates three primary predatory behaviors: prey encirclement, bubble-net feeding maneuvers, and prey search. These behaviors are systematically modeled within the WOA framework.

### 2.1. Prey Encirclement

During this stage, the WOA emulates the actions of humpback whales as they identify and encircle their prey, with the prey representing the potential best answer within the present population. The objective of this stage is to streamline the search process by concentrating efforts around this promising solution. The following equation provides a mathematical representation of this behavior:

(1) $$\begin{array}{c} X\left(t+1\right)=X^{*}-A\cdot D_{1} \end{array}$$

(2) $$\begin{array}{c} D_{1}=\left|C\cdot X^{*}-X\left(t\right)\right| \end{array}$$

where *t* signifies the iteration currently in progress, X(t+1) stands for the position to be searched next, X(t) denotes the present location in the iterative process, and $X^{*}$ signifies the best-found prey position in the ongoing iteration. In the current iteration, the distance designated as $D_{1}$ represents the gap between the whale’s current position X(t) and the prey. The equations for calculating *A* and *C* are provided below:

(3) $$\begin{array}{c} A=2a\cdot r-a \end{array}$$

(4) $$\begin{array}{c} C=2\cdot r \end{array}$$

(5) $$\begin{array}{c} a=2-\frac{2t}{MaxIter} \end{array}$$

where *r* represents randomly generated numbers within the interval $\left[0,1\right]$, and *a* denotes the convergence coefficient, which decreases from 2 to 0 as iterations increase. Furthermore, MaxIter defines the maximum allowable number of iterations, representing the largest possible value for *t*.

### 2.2. Bubble-Net Attack

This phase replicates the upward spiraling feeding behavior of humpback whales during the bubble-net attack. In this stage, the whale advances towards the expected position of the prey in a spiral trajectory. The behavior can be characterized by the following mathematical formula:

(6) $$\begin{array}{c} X\left(t+1\right)=D_{2}\cdot e^{bl}\cdot \cos\left(2\pi l\right)+X^{*} \end{array}$$

(7) $$\begin{array}{c} D_{2}=\left|X^{*}-X\left(t\right)\right| \end{array}$$

where $D_{2}$ represents the current distance between the whale and its target prey, considered the optimal solution. The mathematical representation of the spiral path is constructed using $e^{bl}$ and cos(2πl), where *b* is a constant that determines the spiral’s shape, and *l* is a randomly selected number within the interval $\left[-1,1\right]$.

The mechanisms of encircling and spiral attack are in the process of being refined. During this phase, humpback whales gradually reduce their range of movement as they engage in a spiral hunting pattern. A random number *p*, uniformly distributed between 0 and 1, is employed to decide between the mechanisms, each with an equal probability of 50%. The representation of both scenarios is given by the following equation:

(8) $$\begin{array}{c} X\left(t+1\right)=\left\{\begin{array}{cc} X^{*}-A\cdot D_{1} & \mathrm{if}\;p<0.5\\ D_{2}\cdot e^{bl}\cdot \cos\left(2\pi l\right)+X^{*} & \mathrm{if}\;p\geq 0.5 \end{array}\right. \end{array}$$

### 2.3. Searching for Prey

The phase of exploring prey constitutes a comprehensive search strategy within the WOA framework, aimed at identifying novel possible target zones within the problem’s domain. During this stage, whales arbitrarily pick a search goal and adjust their existing location accordingly. This behavior can be characterized by the following formula:

(9) $$\begin{array}{c} X\left(t+1\right)=X_{rand}\left(t\right)-A\cdot D_{3} \end{array}$$

(10) $$\begin{array}{c} D_{3}=\left|C\cdot X_{rand}\left(t\right)-X\left(t\right)\right| \end{array}$$

In the present iteration, $X_{rand}\left(t\right)$ signifies the location of a whale chosen at random, serving as an arbitrary target, while $D_{3}$ represents the distance separating the present entity from this randomly chosen target. The values of *A* and *C* are determined based on Formulas (3) and (4), respectively. This stage aims to prevent rapid convergence to a suboptimal solution, thereby preserving the diversity of the search process.

The selection of one of the three behavioral strategies is contingent upon the variables *A* and *p*. If *p* is greater than or equal to 0.5, the spiral bubble-net attack is utilized. However, if *p* is less than 0.5 and $\left|A\right|$ is less than 1, encircling prey is the chosen strategy. Alternatively, when $\left|A\right|$ is 1 or greater and *p* is less than 0.5, an extensive search is conducted.

## 3. Proposed ImWOA

In this section, we commence by detailing the three strategies integrated within the proposed ImWOA. Subsequently, we delineate the comprehensive procedure and present the flowchart for the ImWOA. Finally, we provide an analysis of the algorithm’s complexity.

### 3.1. Dynamic Elastic Boundary Optimization Strategy

During the iterative optimization process, certain individuals may exceed the predefined search boundaries. Traditionally, a fixed upper or lower limit is assigned to these individuals. However, this method fails to fully utilize the relevant positional information. Throughout the optimization algorithm’s search process, the entire population should focus on exploring new locations. Meanwhile, the current global optimal solution may represent a potential best position. Improving the boundary handling strategy can effectively enhance the performance of the algorithm and avoid performance degradation when search agents exceed the defined search area.

Drawing from the preceding analysis, we propose the subsequent update rules:

(11) $$\begin{array}{c} x_{i,j}=\left\{\begin{array}{cc} lb_{j}+\alpha \cdot \left(lb_{j}-x_{i,j}\right) & \mathrm{if}\;x_{i,j}<lb_{j}\\ x_{i,j}+\beta \cdot \left(BestPosition_{j}-x_{i,j}\right) & \mathrm{if}\;lb_{j}\leq x_{i,j}\leq ub_{j}\\ ub_{j}-\alpha \cdot \left(x_{i,j}-ub_{j}\right) & \mathrm{if}\;x_{i,j}>ub_{j} \end{array}\right. \end{array}$$

where the j -th dimension’s lower and upper limits are denoted by $lb_{j}$ and $ub_{j}$, respectively. The coordinate indicating the position of the currently optimal individual in the j-th dimension is designated as $BestPosition_{j}$. α and β are stretching factors with values of 0.5 and 0.1, respectively, used to control the intensity of convergence.

The rebound boundary handling method, guided by the optimal value, dynamically adjusts the position of the search individual, thereby ensuring the algorithm’s stability and effectiveness of the algorithm. If the search individual’s position exceeds the boundary, it is redirected back into the boundary and progressively adjusts towards the current optimal position, allowing for more efficient exploration of the search space. This enhancement contributes to improved global search capabilities and accelerates the algorithm’s convergence.

### 3.2. Improved Random Searching Strategy

The random search strategy allows whales to effectively navigate and explore the solution space, which is essential for preserving population diversity. However, during the course of the search, the entire population may be attracted to a randomly selected whale individual. This high level of unpredictability can lead to a reduction in both convergence speed and stability. To tackle this problem, an enhanced random search strategy is proposed, which incorporates the optimal individual and the mean position of all current individuals as the reference target for updating the whales’ positions. This partially mitigates the inherent unpredictability of the original approach and achieves a balance between global and local search capabilities, thereby improving detailed optimization and overall performance.

The following equation provides a representation of the model:

(12) $$\begin{array}{c} x_{i,j}=Bestposition_{j}+W\cdot \left(X_{mean,j}-x_{i,j}\right) \end{array}$$

(13) $$\begin{array}{c} W=1.5\cdot \cos(1.5-\frac{t}{MaxIter})\cdot rand \end{array}$$

where $BestPosition_{j}$ represents the coordinate of the current best individual’s position in the j-th dimension, $X_{mean,j}$ represents the coordinate in the j-th dimension of the mean position of all current individuals, rand is a random number within $\left[0,1\right]$, and MaxIter signifies the maximum number of iterations allowed.

**Figure 1:**
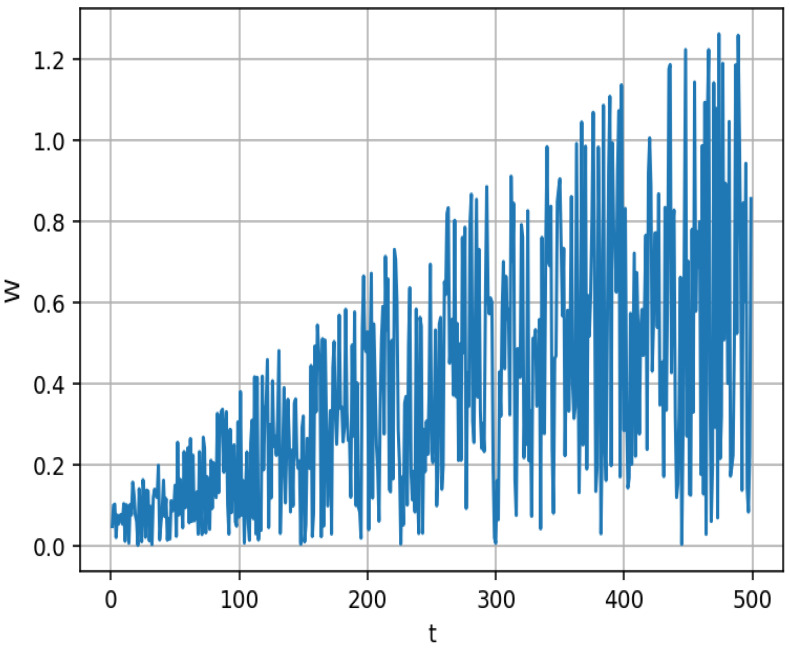
Trajectory diagram of W.

Figure 1 depicts the trajectory of *W*. Analysis of the trend in the figure reveals minimal fluctuation when *t* is small, with a gradual increase in fluctuation amplitude as *t* increases. This observation suggests that the algorithm maintains relative stability during the initial stages and progressively adopts a more exploratory approach in the later stages. A detailed examination of the weights presented in the figure indicates a progressively increasing fluctuation amplitude over time. This pattern signifies that as the optimization process advances, the algorithm’s exploration intensity correspondingly increases, thereby aiding in the avoidance of local optima in the later stages. In conclusion, the incorporation of the cosine function in the weights is designed to facilitate a smooth adjustment, while the element of randomness ensures that the algorithm does not become trapped in local minima.

Integrating the optimal individual’s position into the update rule allows the algorithm to remain flexible and prevent early convergence to suboptimal solutions. Through introducing the mean information of all individuals, it enhances the utilization of other members within the population, which helps expand the search scope and thereby avoids getting trapped in local optima. Combining these factors forms a highly adaptable search mechanism that enables the algorithm to dynamically adjust its search strategy in diverse environments.

### 3.3. Combined Mutation Mechanism

In optimization algorithms, reliance on a single mutation mechanism often leads to entrapment in local optima, thereby limiting diversity within the solution space. This constraint diminishes the algorithm’s search capability and hinders its adaptability to the demands of various complex problems. Therefore, this paper innovatively designs a combined mutation mechanism to improve the algorithm’s overall search capability.

Building on the preceding analysis, we suggest the following update rules:

(14) $$\begin{array}{c} x_{i}=\left\{\begin{array}{cc} x_{i}+\delta_{1} & \mathrm{if}\;case1\\ x_{i}+\delta_{2} & \mathrm{if}\;case2\\ x_{i}+\delta_{3} & \mathrm{if}\;case3 \end{array}\right. \end{array}$$

(15) $$\begin{array}{c} \delta_{1}=\left(rand\left(dim\right)-0.5\right)\cdot \left(ub-lb\right)\cdot 0.1 \end{array}$$

(16) $$\begin{array}{c} \delta_{2}=\left(1-rand^{{\left(1-\frac{t}{MaxIter}\right)}^{2}}\right)\cdot \left(ub-lb\right)\cdot 0.01 \end{array}$$

(17) $$\begin{array}{c} \delta_{3}=\left(1-rand^{{\left(1-\frac{t}{MaxIter}\right)}^{3}}\right)\cdot \left(ub-lb\right)\cdot \left(rand\left(dim\right)-0.5\right)\cdot 0.01 \end{array}$$

where rand(dim) represents a vector of “dim” dimensions with each component being a random value between 0 and 1. The variables lb and ub represent the lower and upper bounds, respectively, both of dimension “dim”. Additionally, rand is a vector of random values in the interval $\left[0,1\right]$, and MaxIter represents the upper limit of iterations. The three mutation scenarios have equal probabilities of occurrence.

By combining these three mutation strategies, the algorithm achieves a balanced equilibrium between exploration and exploitation, thereby enhancing overall optimization performance. The incorporation of multiple mutations during the mutation operation enhances population diversity, prevents entrapment in local optima, and consequently boosts the algorithm’s global search effectiveness.

### 3.4. Whole Framework for ImWOA

**Figure 2:**
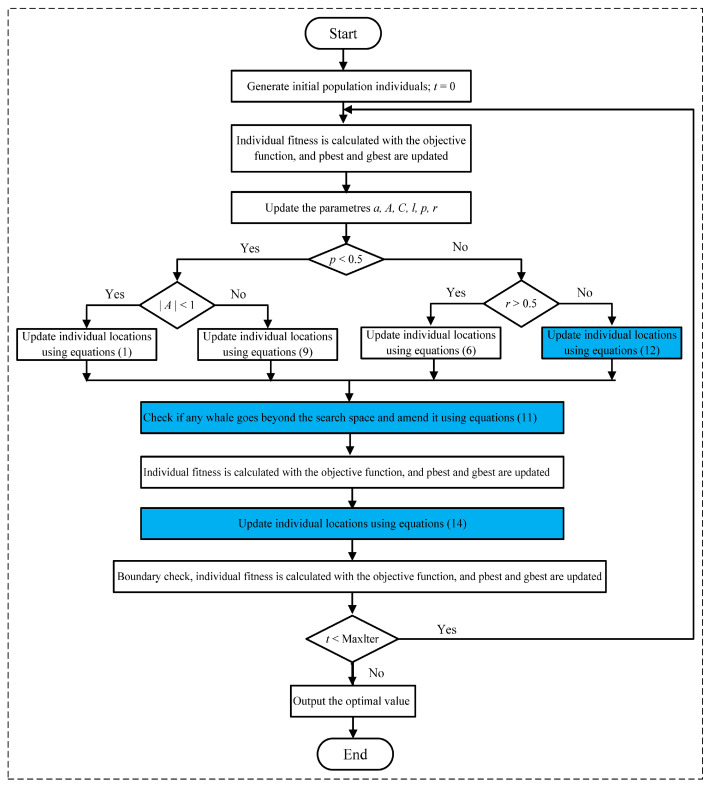
Framework of ImWOA.

The overall structure of the suggested ImWOA approach is depicted in Figure 2.

### 3.5. Computational Complexity Analysis of Algorithms

Time complexity is a crucial metric for evaluating an algorithm’s computational requirements. In the context of the WOA, we designate *N* as the number of whales, *T* as the iteration cap, and D as the problem’s dimensionality. The WOA’s time complexity is formulated as $O\left(N*T*D\right)$. The ImWOA marks an enhancement over its predecessor. An examination of its procedural enhancements— such as dynamic elastic boundary optimization, refined random search techniques, and a combined mutation approach— reveals that these improvements do not augment the algorithm’s overall scale, iteration count, or problem dimensionality. Consequently, the time complexity remains at $O\left(N*T*D\right)$. In summary, ImWOA’s time complexity aligns with that of the standard WOA.

## 4. Experimental Results and Discussions

This research assesses the effectiveness of the improved algorithm using the CEC2017 benchmark functions [34] as the basis for assessment. Detailed specifications of these benchmark functions are presented in Table 1. The CEC2017 dataset comprises 29 benchmark functions designed for single-objective evaluation, with the exception of the original F2 function.

The benchmark functions utilized in this study encompass several categories. Functions F1 and F2 exhibit unimodal characteristics with a distinct global minimum. In contrast, functions F3 through F9 are categorized as basic multimodal functions, characterized by the presence of multiple local minima. Functions F10 through F19 are hybrid in nature, characterized by merging components from three or more CEC2017 benchmark functions via rotation or translation. Finally, functions F20 to F29 represent intricate combinations, consisting of at least three diverse benchmark functions from either a mixed set or the CEC2017 collection. These functions have undergone transformations such as rotation and shifting, thereby increasing their complexity and the challenges associated with solving them.

The algorithms evaluated in this study include CSA, HHO, EHO, BES, WOA, and three WOA variants: ImWOA1, ImWOA2, and ImWOA3. To ensure experimental consistency, we set a uniform initial population of 30 and a fixed max iteration of 500 for all algorithms. To mitigate the effects of randomness, each algorithm was independently run 30 times. The algorithmic parameters are detailed in Table 2.

### 4.1. Impact of Different Strategies on the Algorithm

This section presents a detailed analysis of the rationality and practicality of three distinct strategies and their integration, accompanied by a thorough discussion of their respective impacts on the algorithm. The basic WOA is enhanced by integrating the dynamic elastic boundary optimization strategy, resulting in the approach known as DEBWOA. The improved random searching strategy is incorporated into the approach known as IRSWOA. Furthermore, a combined mutation mechanism is employed in the approach referred to as CMMWOA. For the 50-dimensional scenario, a comparative analysis is conducted with the original WOA and ImWOA using simulation experiments based on CEC2017 benchmark functions. The experimental results, as illustrated in Table 3, provide a clear demonstration of the performance of the strategies under evaluation.

DEBWOA secured the top position on functions F12 and F20, IRSWOA achieved first place on functions F5, F9–F10, F15–F16, F21, and F26, while CMMWOA exhibited strong performance across most cases, notably ranking first on functions F1 and F7–F8. In addressing specific multi-peak problems, DEBWOA demonstrates exceptional proficiency, characterized by its robust local search capabilities. Conversely, IRSWOA exhibits a distinct advantage in resolving multi-peak issues, effectively circumventing entrapment in local optima. CMMWOA, meanwhile, is distinguished by its comprehensive and versatile nature, adeptly managing both single-peak and multi-peak challenges, thereby underscoring its remarkable global search capabilities and adaptability.

The algorithms are ranked based on their average ranking, which is determined by dividing the total cumulative ranking by the number of benchmark functions. Algorithms with the lowest average ranking are considered the top performers, achieving the highest overall performance rating. The data clearly demonstrates that all three enhanced algorithms surpass the original WOA in terms of performance. Among these, the improved random searching strategy exhibits the most significant performance enhancement, followed by the combined mutation mechanism, and finally, the dynamic elastic boundary optimization strategy. When these three strategies are integrated, the ImWOA introduced in this study achieves optimal performance, confirming its efficacy as a multi-strategy improvement algorithm.

### 4.2. Analysis and Results of CEC2017 Test Functions

Our experiments were conducted within a Python 3.10 environment, running the provided source code. We meticulously documented and analyzed the statistical results pertaining to the CEC2017 benchmark functions, addressing both 30-dimensional and 100-dimensional scenarios. These results encompass the minimum (min), average, and standard deviation (Std) values calculated across thirty separate executions of each algorithm. To enhance clarity and emphasize significant findings, the optimal mean result for each benchmark function is emphasized in bold text. Additionally, the “Total” row at the bottom of the Table 4 and Table 5 indicates the frequency with which each algorithm achieved the highest average performance across all tested functions. Detailed statistical reports for the 30-dimensional and 100-dimensional scenarios can be found in Table 4 and Table 5, respectively.

As shown in Table 4, ImWOA demonstrated exceptional performance in the 30-dimensional context, achieving the highest average solution in 25 out of the 29 test functions. For the remaining four functions, although ImWOA did not attain the optimal solution, it demonstrated its competence by producing satisfactory, near-optimal results, thereby underscoring its effectiveness across a diverse range of problems.

Referring to Table 5, the ImWOA exhibited outstanding performance in the 100-dimensional context, achieving the best average solution in 26 out of the 29 test functions. Although it did not achieve optimal results for the remaining three functions, the algorithm still produced notably strong suboptimal outcomes. This highlights ImWOA’s robustness and reliability in tackling high-dimensional problems.

To further clarify these findings, a detailed examination of the results is provided below:

1. For the unimodal functions F1–F2, ImWOA obtained the best average outcomes in both the 30-dimensional and 100-dimensional scenarios, significantly outperforming other algorithms. This demonstrates its powerful optimization capability for unimodal functions.
2. In the 30-dimensional scenario involving the simple multimodal functions F3–F9, ImWOA outperformed all other algorithms by achieving the highest average fitness value across all seven test functions. In the 100-dimensional case, ImWOA achieved optimal average value in five out of seven test functions, with exceptions being F5 and F8. In summary, ImWOA demonstrates superior convergence accuracy and robust global exploration capabilities when applied to simple multimodal functions, making its computational performance highly competitive.
3. In the 30-dimensional scenario, ImWOA excelled in nine out of ten Hybrid functions (F10–F19), achieving the top average fitness value, with the exception of F10, where it ranked second. Similarly, in the 100-dimensional context, ImWOA led in nine out of ten functions, with F18 being the only exception where it ranked second. Overall, ImWOA showcased its proficiency in balancing exploitation and exploration, effectively avoiding local optima and thus providing a significant advantage over other algorithms in tackling Hybrid functions.
4. For the Composition functions (F20–F29), in the 30-dimensional scenario, ImWOA achieved the optimal average results for 10 test functions except for F26 and F28–F29, where it still secured the second-best performance. In the 100-dimensional context, ImWOA attained the best rank for all 10 test functions, far surpassing other algorithms. Overall, for the Composition functions, ImWOA demonstrated exceptional optimization capabilities and robust performance, especially in high-dimensional spaces, offering significant advantages compared to other algorithms.

### 4.3. Analysis of the Convergence Behavior of the Algorithms

**Figure 3:**
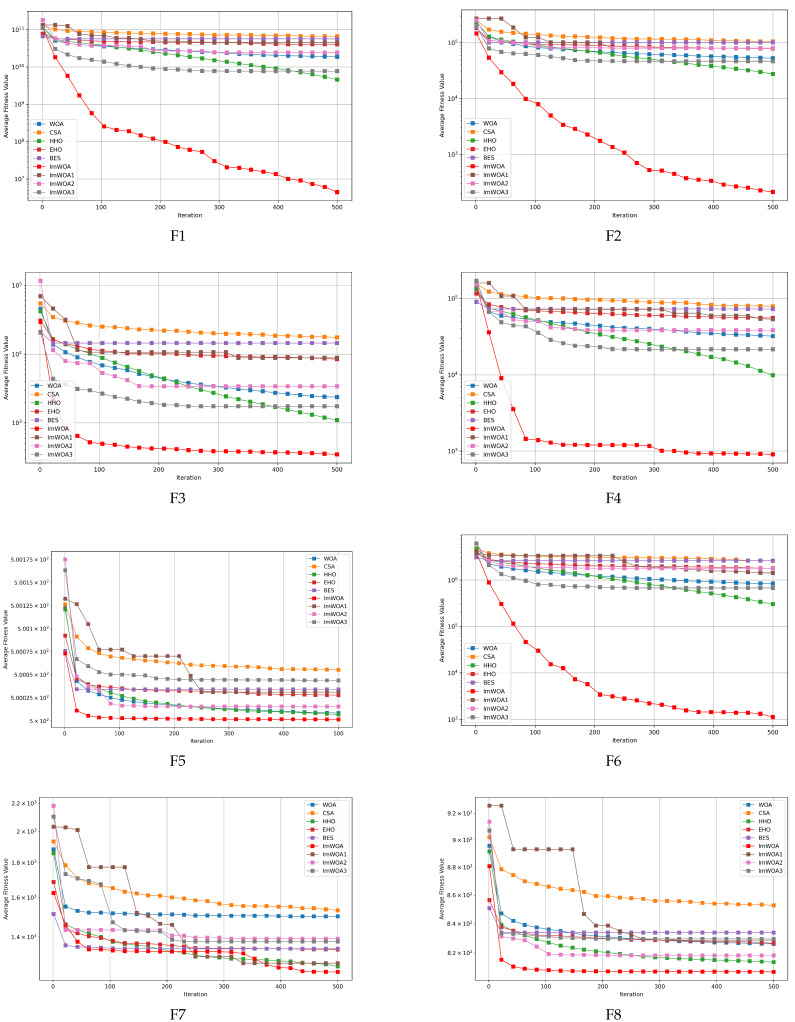

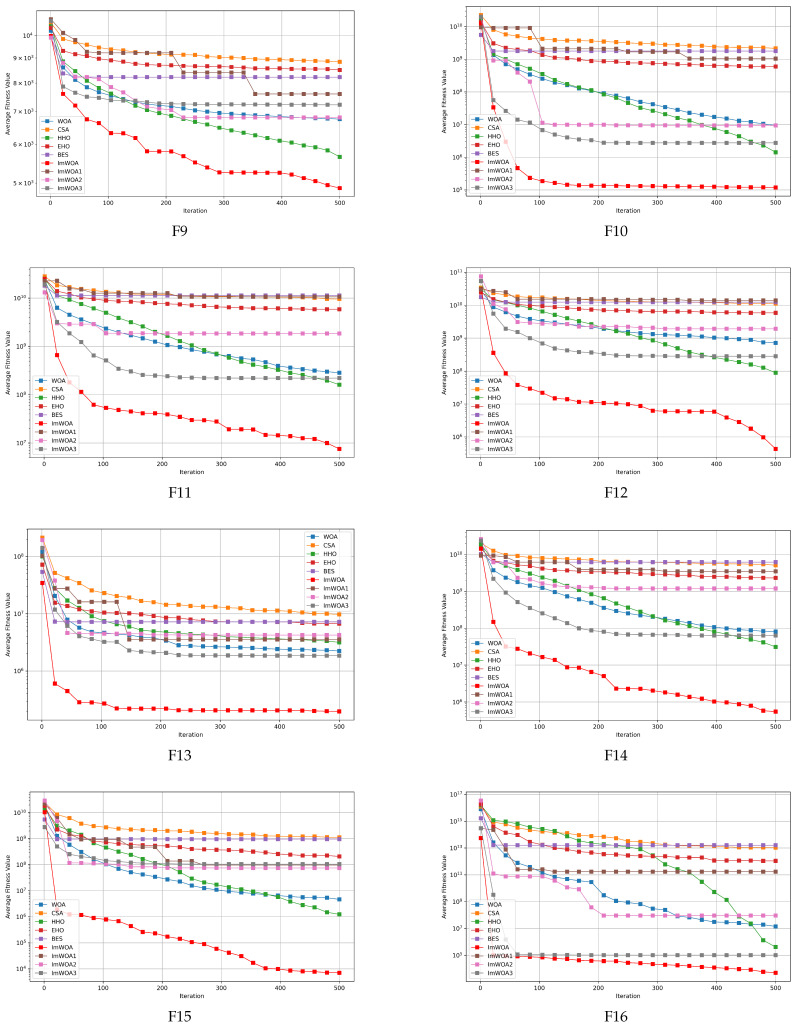

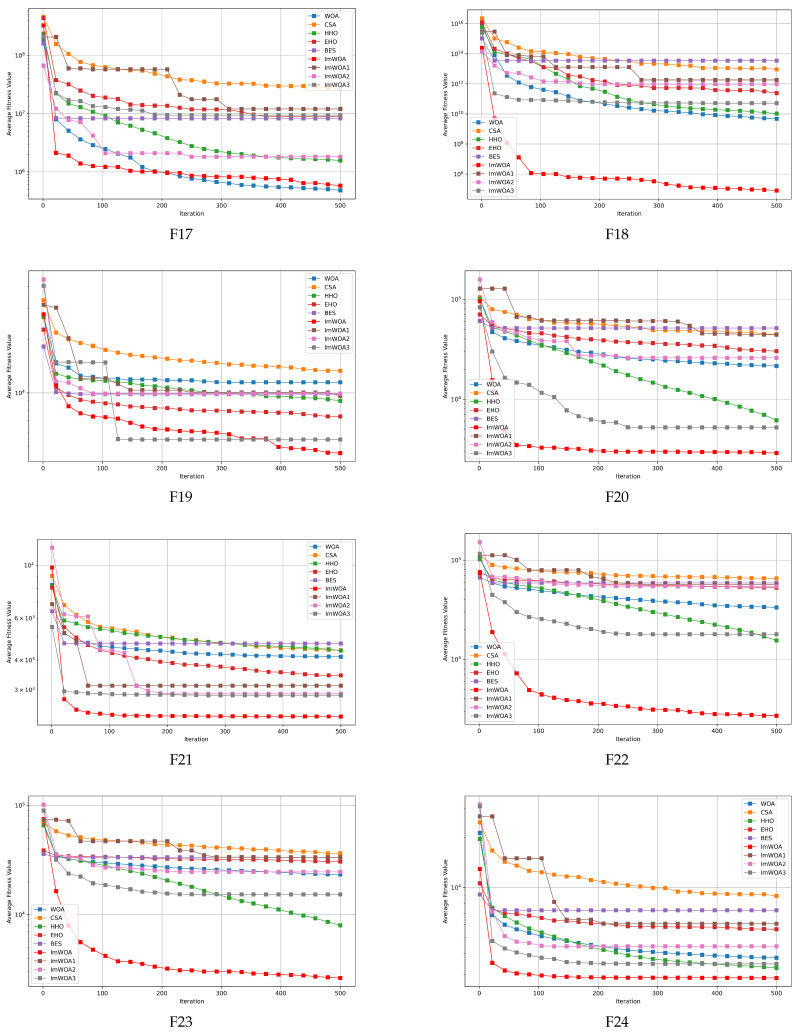

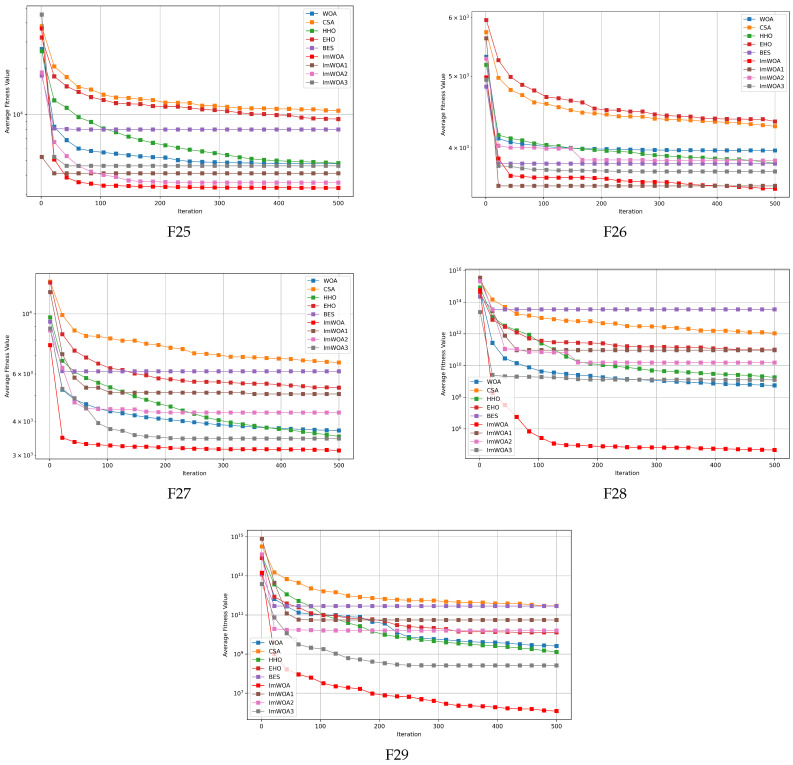
CEC2017 test curves chart (Dim = 30).

**Figure 4:**
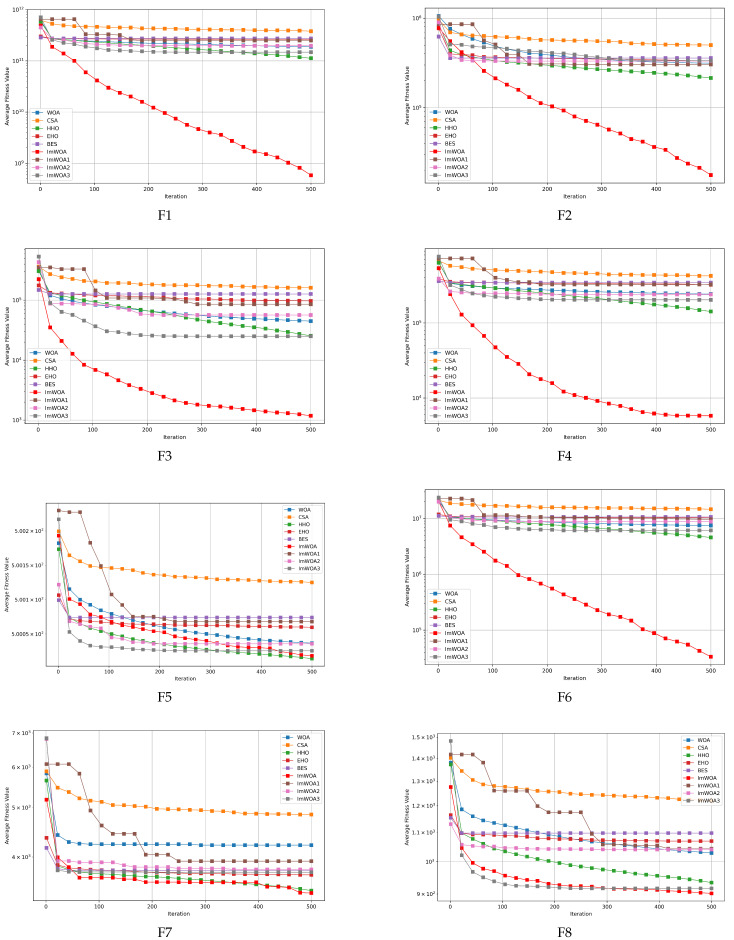

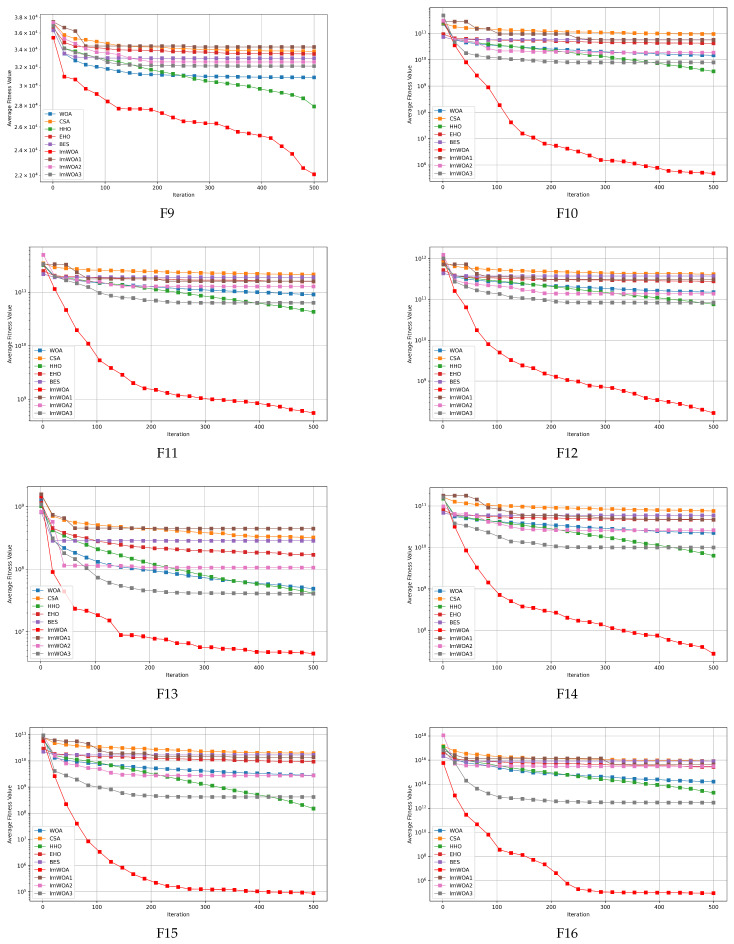

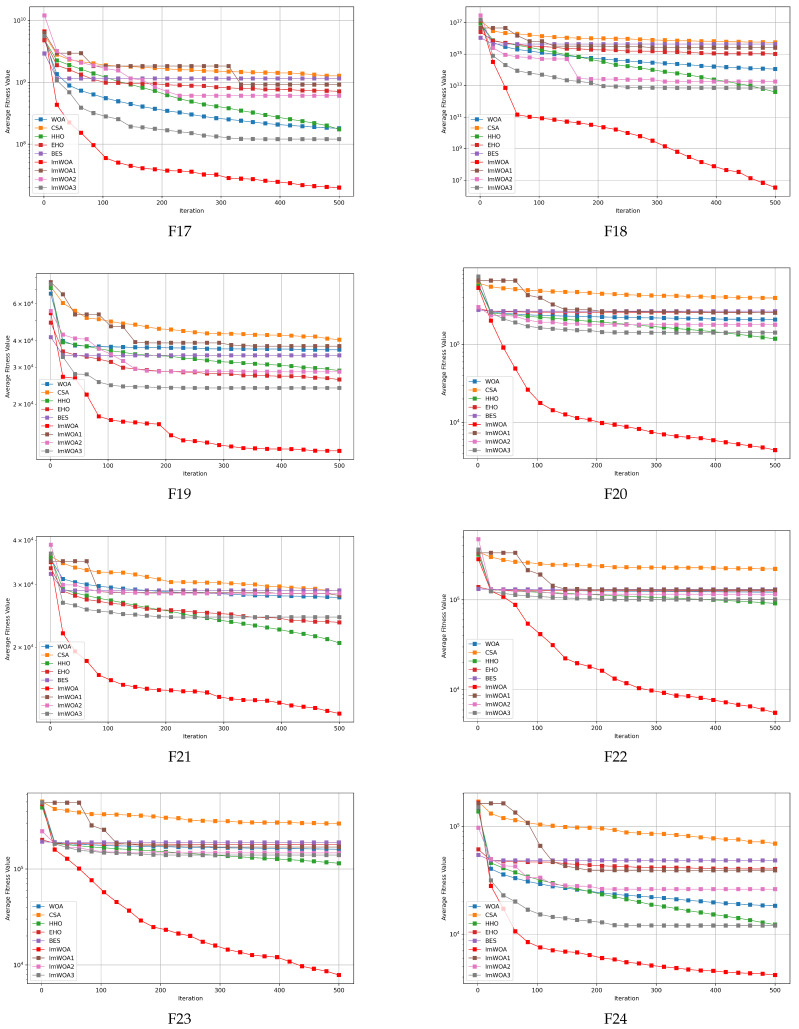

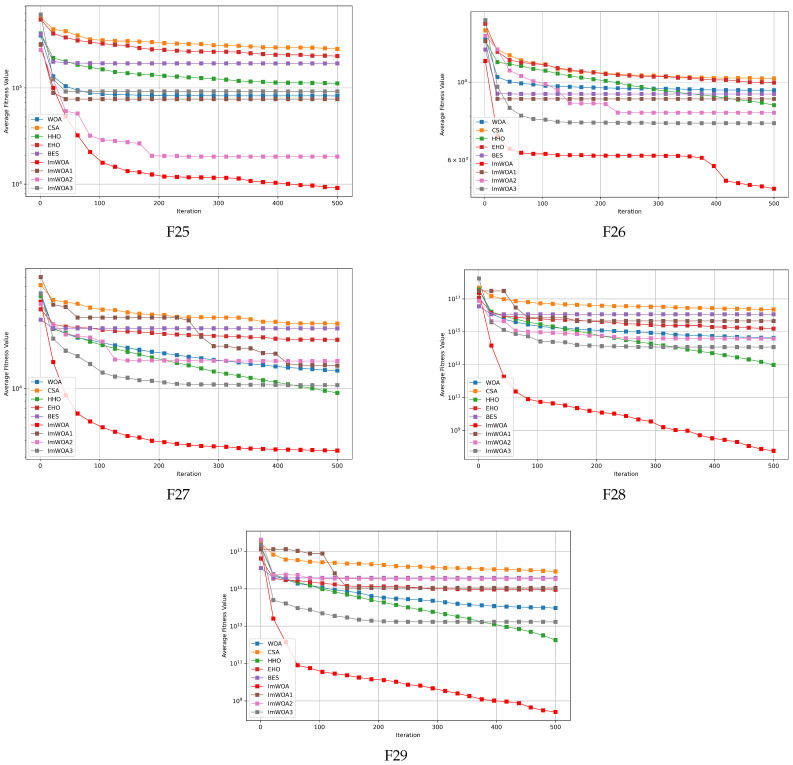
CEC2017 test curves chart (Dim = 100).

To assess the algorithms’ convergence accuracy and rate, convergence graphs were generated for scenarios involving both 30 and 100 dimensions, showcasing ImWOA’s performance in comparison with other algorithms, as depicted in Figure 3 and Figure 4. In these figures, each subplot presents the iteration count on the x-axis and the mean convergence trend of the function test results on the y-axis, derived from 30 independent trials. Upon reviewing these graphs, the following conclusions can be drawn:

1. With respect to the unimodal problems F1–F2, ImWOA consistently demonstrated excellent performance in terms of convergence rate and precision, outperforming all other tested algorithms in both the 30-dimensional and 100-dimensional contexts.
2. In relation to the simple multimodal functions F3–F9, ImWOA demonstrated a significantly faster convergence compared to other algorithms on the remaining test functions, with marginally better performance in certain benchmarks. Importantly, ImWOA’s convergence speed surpassed that of WOA across these functions, highlighting the effectiveness of the enhancement strategy. In the 100-dimensional context, ImWOA again exhibited rapid convergence on F3–F4, F6, and F9, consistently outperforming WOA in terms of convergence speed on additional test functions, further affirming the success of the improvement strategy.
3. Regarding the hybrid functions ranging from F10 to F19, ImWOA showcased a remarkably swift convergence rate in the 30-dimensional scenario, especially for F10–F16 and F18, achieving significant superiority over other algorithms. In the 100-dimensional scenario, ImWOA’s convergence speed was extremely fast for all test functions, markedly surpassing other methods. This highlights ImWOA’s superior optimization performance and its efficiency in high-dimensional spaces. Overall, ImWOA exhibited exceptional results for the hybrid functions.
4. In the context of composite functions F20–F29, within a 30-dimensional framework, ImWOA exhibited extremely rapid convergence rates on functions F20–F24 and F27–F29, far surpassing other algorithms. Additionally, ImWOA demonstrated a faster convergence speed compared to WOA across the remaining test functions, thereby substantiating the effectiveness of the improvement strategy. In the 100-dimensional case, ImWOA’s performance was even more noteworthy, with its convergence rate significantly exceeding that of all other algorithms. This highlights ImWOA’s exceptionally robust convergence speed and underscores its stability and excellence in high-dimensional spaces.

### 4.4. Wilcoxon Rank-Sum Test

The Wilcoxon rank-sum test, acknowledged for its robustness as a non-parametric statistical method, was utilized to assess performance differences between enhanced and conventional algorithms, eliminating the requirement for presumptions about particular data distributions. In this study, this technique was applied to examine distinctions among five benchmark algorithms, three WOA variants, and ImWOA. Through comprehensive testing on the CEC2017 dataset across multiple dimensions, we gained valuable insights into the performance characteristics of various algorithms for solving optimization challenges.

A *p*-value less than 0.05 suggests rejection of the null hypothesis, indicating that the two algorithms differ significantly in a statistical sense. Conversely, a *p*-value greater than 0.05 suggests that the two algorithms perform similarly. Table 6 and Table 7 display the Wilcoxon rank-sum test results comparing ImWOA with other algorithms, with *p*-values below 0.05 highlighted in bold. An examination of Table 6 and Table 7 reveals that ImWOA demonstrates a notable distinction from the other algorithms. Specifically, in both 30-dimensional and 100-dimensional spaces, the *p*-values for ImWOA in comparison to all other algorithms across all test functions are predominantly below 0.05, indicating statistical significance. In conclusion, ImWOA shows a clear superiority over all other algorithms, with robust statistical evidence supporting for this advantage.

### 4.5. Friedman Test

**Figure 5:**
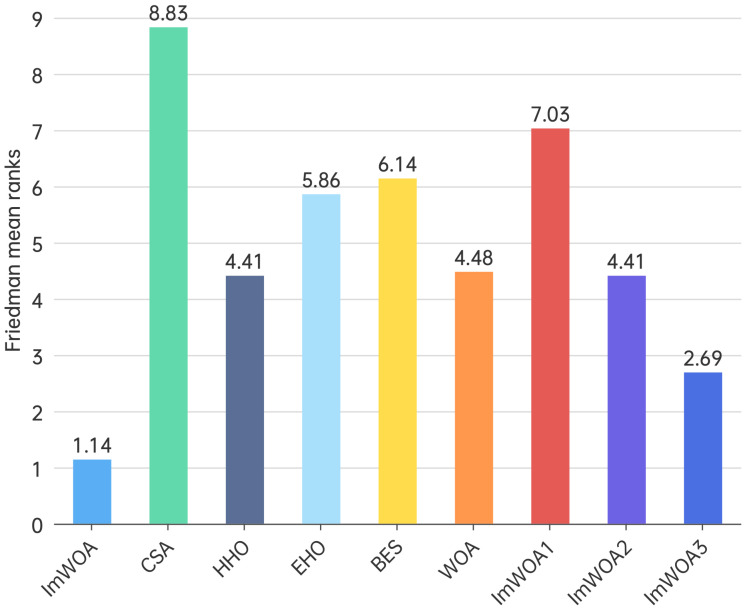
Friedman mean ranks obtained by the employed algorithms (30 dim).

**Figure 6:**
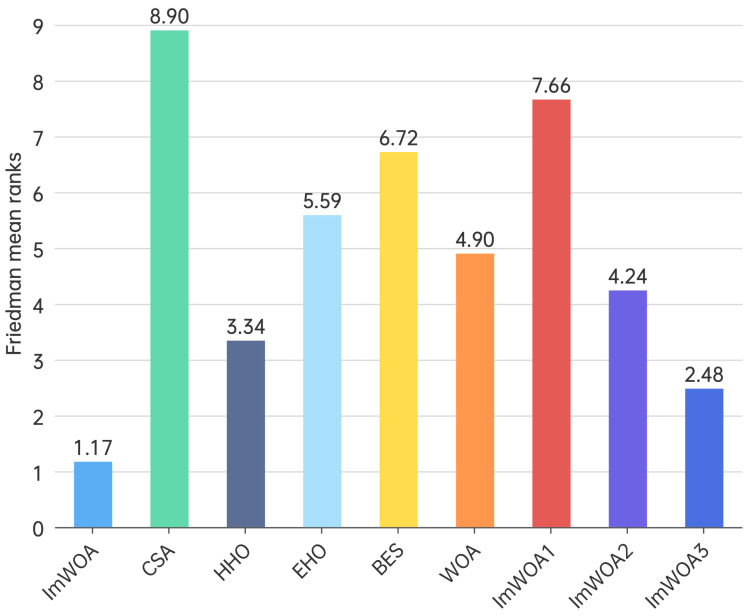
Friedman mean ranks obtained by the employed algorithms (100 dim).

The Friedman test is a non-parametric statistical method used for comparing three or more groups, particularly when dealing with non-normally distributed data or small sample sizes. Similar to the Kruskal–Wallis test, it is appropriate for repeated measures or block design experiments. This test evaluates differences by comparing the mean ranks across groups to identify statistically significant variations. Figure 5 and Figure 6 illustrate the average ranking outcomes of the algorithms assessed using the CEC2017 benchmark functions. The results clearly indicate that ImWOA achieved the highest rank, followed sequentially by ImWOA3, HHO, ImWOA2, WOA, EHO, BES, ImWOA1, and CSA.

### 4.6. Sensitivity of ImWOA to Parameter Variations

In the operational framework of the ImWOA algorithm, the decision to execute either a bubble-net attack or an enhanced random search strategy is contingent upon whether a random number *r* within the range of 0 to 1 exceeds the threshold of 0.5. This threshold, denoted as *k*, is a critical parameter influencing the algorithm’s trade-off between searching widely and refining solutions. To examine the sensitivity of ImWOA to changes in this parameter, we conducted an analysis by varying *k* from its original value of 0.5 to 0.4 and 0.6. The effectiveness of the algorithm was then assessed across the CEC2017 test functions in a 100-dimensional space.

The detailed results are presented in Table 8. The mean ranking is calculated by dividing the sum of all individual rankings by the count of benchmark functions. Algorithms are then ordered based on this mean, with those exhibiting the lowest average ranking deemed the top performers and thus receiving the highest combined performance ranking. Table 8 indicates that ImWOA attains the best average ranking when *k* = 0.5, followed by the second-best average ranking when *k* = 0.6, and the poorest average ranking when *k* = 0.4.

## 5. Engineering Design Problems

To further validate ImWOA’s applicability in real-world engineering contexts, we assess its optimization capabilities across diverse scenarios using three practical engineering design challenges: reducer design, vehicle side impact design, and welded beam design. These design problems are optimization tasks that involve constraints on their variables, which must be addressed.

There are three primary strategies for managing constraints: employing a penalty function, adhering to a feasibility rule, or utilizing a multi-objective approach. In this study, constraints are transformed into penalty functions using the external penalty function method, which are then integrated with the primary objective function. By setting the penalty parameter to 100,000, we ensure that any constraint violations during the optimization process are penalized, facilitating the identification of an optimal solution that adheres to all constraints.

The algorithms evaluated in this research include CSA, HHO, EHO, BES, WOA, and three variants of WOA: ImWOA1, ImWOA2, and ImWOA3. To ensure fairness in our experiments, we standardized the initial population at 30 and set a maximum iteration limit of 500 for all algorithms. To mitigate the influence of randomness, each algorithm was executed independently 30 times, and we calculated the mean performance and consistency of their results. This approach provides a comprehensive assessment, taking into account both the overall effectiveness and reliability, which is essential for evaluating the applicability of these algorithms in practical engineering contexts.

### 5.1. Reducer Design Problem

Designing a speed reducer requires consideration of seven parameters: the end face width ($x_{1}$), teeth module ($x_{2}$), pinion teeth count ($x_{3}$), length of the first shaft segment between bearings ($x_{4}$), length of the second shaft segment between bearings ($x_{5}$), diameter of the first shaft ($x_{6}$), and diameter of the second shaft ($x_{7}$). The objective is to minimize the overall weight of the gearbox by optimizing these parameters. The target function is articulated in Equation (18), while the constraints are detailed in Equation (19). Equation (20) establishes the variable bounds.

Minimize:

(18) $$\begin{array}{cc} \;f(x)= & 0.7854x_{1}x_{2}^{2}\left(3.3333x_{3}^{2}+14.9334x_{3}-43.0934\right)-1.508x_{1}\left(x_{6}^{2}+x_{7}^{2}\right)\\ \; & +7.4777x_{6}^{3}+x_{7}^{3}+0.7854x_{4}x_{6}^{2}+x_{5}x_{7}^{2} \end{array}$$

Subject to:

(19) $$\begin{array}{cc} \; & g_{1}\left(x\right)=\frac{27}{x_{1}x_{2}^{2}x_{3}}-1\leq 0\\ \; & g_{2}\left(x\right)=\frac{397.5}{x_{1}x_{2}^{2}x_{3}^{2}}-1\leq 0\\ \; & g_{3}\left(x\right)=\frac{1.93x_{4}^{3}}{x_{2}x_{3}x_{6}^{4}}-1\leq 0\\ \; & g_{4}\left(x\right)=\frac{1.93x_{5}^{3}}{x_{2}x_{3}x_{7}^{4}}-1\leq 0\\ \; & g_{5}\left(x\right)=\frac{1}{110x_{6}^{3}}\times \sqrt{{\left(\frac{745x_{4}}{x_{2}x_{3}}\right)}^{2}+16.9\times 10^{6}}-1\leq 0\\ \; & g_{6}\left(x\right)=\frac{1}{85x_{7}^{3}}\times \sqrt{{\left(\frac{745x_{5}}{x_{2}x_{3}}\right)}^{2}+16.9\times 10^{6}}-1\leq 0\\ \; & g_{7}\left(x\right)=\frac{x_{2}x_{3}}{40}-1\leq 0\\ \; & g_{8}\left(x\right)=\frac{5x_{2}}{x_{1}}-1\leq 0\\ \; & g_{9}\left(x\right)=\frac{x_{1}}{12x_{2}}-1\leq 0\\ \; & g_{10}\left(x\right)=\frac{1.5x_{6}+1.9}{x_{4}}-1\leq 0\\ \; & g_{11}\left(x\right)=\frac{1.1x_{7}+1.9}{x_{5}}-1\leq 0 \end{array}$$

Parameters range:

(20) $$\begin{array}{cc} \; & 2.6\leq x_{1}\leq 3.6\\ \; & 0.7\leq x_{2}\leq 0.8\\ \; & 17\leq x_{3}\leq 28\\ \; & 7.3\leq x_{4}\leq 8.3\\ \; & 7.3\leq x_{5}\leq 8.3\\ \; & 2.9\leq x_{6}\leq 3.9\\ \; & 5.0\leq x_{7}\leq 5.5 \end{array}$$

Table 9 displays the experimental findings pertaining to the reducer design challenge. The top-ranked average result is highlighted in bold text. The average results indicate that ImWOA significantly surpasses other algorithms, highlighting its effectiveness in delivering precise solutions and showcasing its remarkable stability and robustness.

### 5.2. Vehicle Side Impact Design Problem

The objective of vehicle side impact design is to reduce vehicle mass while maintaining optimal performance. This challenge encompasses 11 decision variables and 10 constraint conditions. These decision variables include the thickness of several components: the B-pillar inner panel ($x_{1}$), B-pillar reinforcement ($x_{2}$), inner floor ($x_{3}$), crossbeam ($x_{4}$), door beam ($x_{5}$), door beltline reinforcement ($x_{6}$), and roof longitudinal beam ($x_{7}$). Additionally, the decision variables account for the carbon content of the materials used for the inner side of the B-pillar ($x_{8}$) and the inner side of the floor ($x_{9}$), as well as the height of the guardrail ($x_{10}$) and the collision position ($x_{11}$). The objective function is defined in Equation (21), with the associated constraints detailed in Equation (22). Equation (23) specifies the bounds for each variable.

Minimize:

(21) $$\begin{array}{c} \;f\left(x\right)=1.98+4.9x_{1}+6.67x_{2}+6.98x_{3}+4.01x_{4}+1.78x_{5}+2.73x_{7} \end{array}$$

Subject to:

(22) $$\begin{array}{cc} \; & g_{1}\left(x\right)=1.16-0.3717x_{2}x_{4}-0.484x_{3}x_{9}+0.01343x_{6}x_{10}-1\leq 0\\ \; & g_{2}\left(x\right)=0.261-0.0159x_{1}x_{2}-0.188x_{1}x_{8}+0.019x_{2}x_{7}\\ \; & \;\;\;+0.0144x_{3}x_{5}+0.0008757x_{5}x_{10}+0.080405x_{6}x_{9}\\ \; & \;\;\;+0.00139x_{8}x_{11}+0.00001575x_{10}x_{11}-0.32\leq 0\\ \; & g_{3}\left(x\right)=0.214+0.00817x_{5}-0.131x_{1}x_{8}-0.0704x_{1}x_{9}+0.03099x_{2}x_{6}\\ \; & \;\;\;-0.018x_{2}x_{7}+0.0208x_{3}x_{8}+0.121x_{3}x_{9}-0.00364x_{5}x_{6}\\ \; & \;\;\;+0.0007715x_{5}x_{10}-0.0005354x_{6}x_{10}+0.00121x_{8}x_{1}-0.32\leq 0\\ \; & g_{4}\left(x\right)=0.074-0.061x_{2}-0.163x_{3}x_{8}+0.001232x_{3}x_{10}\\ \; & \;\;\;-0.166x_{7}x_{9}+0.227x_{2}^{2}-0.32\leq 0\\ \; & g_{5}\left(x\right)=28.98+3.818x_{3}-4.2x_{1}x_{2}+0.0207x_{5}x_{10}\\ \; & \;\;\;+6.63x_{6}x_{9}-7.7x_{7}x_{8}+0.32x_{9}x_{10}-32\leq 0\\ \; & g_{6}\left(x\right)=33.86+2.95x_{3}+0.1792x_{10}-5.057x_{1}x_{2}-11x_{2}x_{8}\\ \; & \;\;\;-0.0215x_{5}x_{10}-9.98x_{7}x_{8}+22x_{8}x_{9}-32\leq 0\\ \; & g_{7}\left(x\right)=46.36-9.9x_{2}+12.9x_{1}x_{8}+0.1107x_{3}x_{10}-32\leq 0\\ \; & g_{8}\left(x\right)=4.72-0.5x_{4}-0.19x_{2}x_{3}-0.0122x_{4}x_{10}\\ \; & \;\;\;+0.009325x_{6}x_{10}+0.000191x_{11}^{2}-4\leq 0\\ \; & g_{9}\left(x\right)=10.58-0.674x_{1}x_{2}-1.95x_{2}x_{8}+0.02054x_{3}x_{10}\\ \; & \;\;\;-0.0198x_{4}x_{10}+0.028x_{6}x_{10}-9.9\leq 0\\ \; & g_{10}\left(x\right)=16.45-0.489x_{3}x_{7}-0.843x_{5}x_{6}+0.0432x_{9}x_{10}\\ \; & \;\;\;-0.0556x_{9}x_{11}+0.000786x_{11}^{2}-15.7\leq 0 \end{array}$$

Parameters range:

(23) $$\begin{array}{cc} \; & 0.5\leq x_{1},x_{2},x_{3},x_{4},x_{5},x_{6},x_{7}\leq 1.5\\ \; & \;\;\;0\leq x_{8},x_{9}\leq 1\\ \; & \;-30\leq x_{10},x_{11}\leq 30 \end{array}$$

Table 10 presents the optimization outcomes concerning the design issues of vehicle side impacts. The top-ranked average result is highlighted in bold text. Notably, ImWOA attains the lowest average manufacturing cost, outperforming other algorithms and demonstrating its efficacy in this specific optimization context.

### 5.3. Welded Beam Design Problem

The primary objective in tackling the welded beam design challenge is to reduce the beam’s cost to a minimum. This involves optimizing four key variables: weld thickness (*h*), length of the connected bar segment (*l*), bar height (*t*), and thickness of the reinforcing bar (*b*), as described in Equation (24). The goal is to find the minimum value of the function presented in Equation (25), while adhering to constraints such as shear stresses (τ), beam bending stresses (θ), bar flexural loads ($P_{c}$), and beam end deflections (δ), as specified in Equation (26). These variables are subject to the constraints outlined in Equation (27), and the relevant parameter values and their respective solutions are provided in Equation (28).

Consider:

(24) $$\begin{array}{c} \;x=\left[x_{1},x_{2},x_{3},x_{4}\right]=\left[h,l,t,b\right] \end{array}$$

Minimize:

(25) $$\begin{array}{c} \;f\left(x\right)=1.10471x_{1}^{2}x_{1}+0.04811x_{3}x_{4}\left(14+x_{2}\right) \end{array}$$

Subject to:

(26) $$\begin{array}{cc} \; & g_{1}\left(x\right)=\tau \left(x\right)-\tau_{max}\leq 0\\ \; & g_{2}\left(x\right)=\sigma \left(x\right)-\sigma_{max}\leq 0\\ \; & g_{3}\left(x\right)=\delta \left(x\right)-\delta_{max}\leq 0\\ \; & g_{4}\left(x\right)=x_{1}-x_{4}\leq 0\\ \; & g_{5}\left(x\right)=P-P_{c}\left(x\right)\leq 0\\ \; & g_{6}\left(x\right)=0.125-x_{1}\leq 0\\ \; & g_{7}\left(x\right)=1.10471x_{1}^{2}\\ \; & +0.04811x_{3}x_{4}\left(14+x_{2}\right)-5\leq 0 \end{array}$$

Parameters range:

(27) $$\begin{array}{cc} \; & 0.1\leq x_{1}\leq 2.0\\ \; & 0.1\leq x_{2}\leq 10\\ \; & 0.1\leq x_{3}\leq 10\\ \; & 0.1\leq x_{3}\leq 2.0 \end{array}$$

where

(28) $$\begin{array}{cc} \; & \tau \left(x\right)=\sqrt{{\left(\tau^{\prime }\right)}^{2}+2\tau^{\prime }\tau^{\prime \prime }\frac{x_{2}}{2R}+\left(\tau^{\prime \prime }\right)}\\ \; & \tau^{\prime }=\frac{P}{\sqrt{2x_{1}x_{2}}},\tau^{\prime \prime }=\frac{MR}{J}\\ \; & R=\sqrt{\frac{x_{2}^{2}}{4}+{\left(\frac{x_{1}+x_{3}}{2}\right)}^{2}}\\ \; & \sigma \left(x\right)=\frac{6PL}{x_{4}x_{3}^{2}},M=P\left(L+\frac{x_{2}}{2}\right),\delta \left(x\right)=\frac{6PL^{3}}{Ex_{4}x_{3}^{2}}\\ \; & J=2\left\{\sqrt{2x_{1}x_{2}}\left[\frac{x_{2}^{2}}{4}+{\left(\frac{x_{1}+x_{3}}{2}\right)}^{2}\right]\right\}\\ \; & P_{c}\left(x\right)=\frac{4.013E\sqrt{\frac{x_{3}^{2}x_{4}^{6}}{36}}}{L^{2}}\left(1-\frac{x_{3}}{2L}\sqrt{\frac{E}{4G}}\right)\\ \; & P=6000lb,L=14in,\delta_{max}=0.25in\\ \; & E=30\times 10^{6}psi,\tau_{max}=13600psi,\sigma_{max}=30000psi \end{array}$$

Table 11 displays the outcomes of optimizing the welded beam design. The top-ranked average result is highlighted in bold text. Notably, ImWOA achieves the minimum average manufacturing cost, surpassing other algorithms and demonstrating its efficacy in this specific optimization context.

## 6. Conclusions

This paper presents an advanced algorithm, ImWOA, which integrates three innovative techniques to enhance optimization performance. Firstly, the dynamic elastic boundary optimization strategy is employed to prevent performance decline when solutions extend beyond the predefined search space, while simultaneously improving search efficiency by guiding solutions towards the current best solution. Secondly, an enhanced random search strategy is utilized, which focuses on both the present best solution and the average position of all solutions. This method effectively balances global and local search, ensuring diversity and comprehensive exploration of the search space. Finally, a hybrid mutation mechanism is incorporated to enhance population diversity and minimize the risk of local optima convergence, thus enhancing the algorithm’s overall search capability and computational efficiency. These improvements overcome the original WOA algorithm’s limitations, broadening its search capabilities and adaptability, and leading to more efficient and precise optimization results.

We evaluated ImWOA’s efficacy using the CEC2017 benchmark suite, comparing it with five established algorithms and three WOA variants. ImWOA demonstrated exceptional performance, achieving the highest average outcomes in 25 out of the 29 test functions within a 30-dimensional (30D) context, and obtaining the most favorable mean solutions in 26 out of 29 tests in a 100-dimensional (100D) setting. Furthermore, we validated ImWOA’s capabilities through its application to three engineering challenges: reducer design, vehicle side impact design, and welded beam design. The findings underscored ImWOA’s exceptional performance and its applicability to practical scenarios. These empirical evaluations and real-world case studies highlight ImWOA’s superior performance in both theoretical benchmarks and its practical utility in tackling intricate optimization challenges in engineering domains, signifying its extensive potential and versatility.

Looking forward, there are several pathways for enhancing ImWOA’s performance. One promising direction involves refining the parameter selection and tuning methodologies to improve ImWOA’s capability in addressing large-scale and complex problems. Another approach involves developing hybrid algorithms that integrate the strengths of various swarm intelligence techniques, thereby augmenting optimization efficiency and precision. Additionally, extending the application of ImWOA to a broader range of practical engineering challenges will further validate its versatility and efficacy across diverse domains. These future endeavors hold the promise of uncovering new possibilities and expand the horizons of ImWOA’s potential.

## Figures and Tables

**Table 1:** CEC2017 functions.

Type	No.	Function	Optimal
Unimodal functions	1	Shifted and Rotated Bent Cigar Function	100
	2	Shifted and Rotated Zakharov Function	200
Simple multimodal functions	3	Shifted and Rotated Rosenbrock’s Function	300
	4	Shifted and Rotated Rastrigin’s Function	400
	5	Shifted and Rotated Expanded Scaffer’s F6 Function	500
	6	Shifted and Rotated Lunacek Bi_Rastrigin Function	600
	7	Shifted and Rotated Noncontinuous Rastrigin’s Function	700
	8	Shifted and Rotated Levy Function	800
	9	Shifted and Rotated Schwefel’s Function	900
Hybrid functions	10	Hybrid Function 1 (N = 3)	1000
	11	Hybrid function 2 (N = 3)	1100
	12	Hybrid function 3 (N = 3)	1200
	13	Hybrid function 4 (N = 4)	1300
	14	Hybrid function 5 (N = 4)	1400
	15	Hybrid function 6 (N = 4)	1500
	16	Hybrid function 6 (N = 5)	1600
	17	Hybrid function 6 (N = 5)	1700
	18	Hybrid function 6 (N = 5)	1800
	19	Hybrid function 6 (N = 6)	1900
Composition functions	20	Composition Function 1 (N = 3)	2000
	21	Composition function 2 (N = 3)	2100
	22	Composition function 3 (N = 4)	2200
	23	Composition function 4 (N = 4)	2300
	24	Composition function 5 (N = 5)	2400
	25	Composition function 6 (N = 5)	2500
Composition functions	26	Composition function 7 (N = 6)	2600
	27	Composition function 7 (N = 6)	2700
	28	Composition function 9 (N = 3)	2800
	29	Composition function 10 (N = 3)	2900
Composition functions	20	Composition Function 1 (N = 3)	2000
	21	Composition function 2 (N = 3)	2100
	22	Composition function 3 (N = 4)	2200
	23	Composition function 4 (N = 4)	2300
	24	Composition function 5 (N = 5)	2400
	25	Composition function 6 (N = 5)	2500
	26	Composition function 7 (N = 6)	2600
	27	Composition function 7 (N = 6)	2700
	28	Composition function 9 (N = 3)	2800
	29	Composition function 10 (N = 3)	2900

Search range: [−100, 100].

**Table 2:** Algorithm parameters.

Algorithms	Parameters
ImWOA	b = 1, c = 2· rand, a = 2−$\frac{2t}{MaxIter}$, A = 2· a· r−a, α = 0.5, β = 0.1
CSA [4]	Referring to the literature [4]
HHO [6]	Referring to the literature [6]
EHO [7]	Referring to the literature [7]
BES [5]	Referring to the literature [5]
WOA [8]	b = 1, c = 2· rand, a = 2−$\frac{2t}{MaxIter}$, A = 2· a· r−a
ImWOA1 [35]	Referring to the literature [35]
ImWOA2 [36]	Referring to the literature [36]
ImWOA3 [10]	Referring to the literature [10]

**Table 3:** Impact of different strategies on the algorithm.

Dim = 50						
Func.	Index	WOA	DEBWOA	IRSWOA	CMMWOA	ImWOA
F1	Min	2.49 × 10^10^	3.52 × 10^10^	8.82 × 10^9^	2.07 × 10^8^	2.91 × 10^7^
	Mean	3.54 × 10^10^	4.34 × 10^10^	2.07 × 10^10^	9.94 × 10^9^	9.99 × 10^9^
	Std	2.57 × 10^10^	2.15 × 10^10^	2.76 × 10^10^	2.78 × 10^10^	3.02 × 10^10^
	Rank	4	5	3	1	2
F2	Min	1.76 × 10^5^	1.52 × 10^5^	2.16 × 10^4^	9.73 × 10^3^	4.14 × 10^2^
	Mean	2.79 × 10^5^	1.83 × 10^5^	6.66 × 10^4^	9.51 × 10^4^	2.82 × 10^4^
	Std	1.03 × 10^5^	5.35 × 10^4^	7.69 × 10^4^	1.02 × 10^5^	5.73 × 10^4^
	Rank	5	4	2	3	1
F3	Min	6.74 × 10^3^	3.23 × 10^3^	2.82 × 10^3^	7.07 × 10^2^	5.34 × 10^2^
	Mean	1.12 × 10^4^	6.99 × 10^3^	8.71 × 10^3^	3.45 × 10^3^	2.77 × 10^3^
	Std	1.30 × 10^4^	1.01 × 10^4^	1.89 × 10^4^	7.95 × 10^3^	8.29 × 10^3^
	Rank	5	3	4	2	1
F4	Min	2.59 × 10^4^	2.17 × 10^4^	2.29 × 10^4^	5.48 × 10^3^	1.60 × 10^3^
	Mean	3.79 × 10^4^	3.21 × 10^4^	3.67 × 10^4^	1.85 × 10^4^	1.07 × 10^4^
	Std	2.62 × 10^4^	2.58 × 10^4^	3.06 × 10^4^	3.29 × 10^4^	2.63 × 10^4^
	Rank	5	3	4	2	1
F5	Min	5.00 × 10^2^	5.00 × 10^2^	5.00 × 10^2^	5.00 × 10^2^	5.00 × 10^2^
	Mean	5.00 × 10^2^	5.00 × 10^2^	5.00 × 10^2^	5.00 × 10^2^	5.00 × 10^2^
	Std	2.69 × 10^−2^	1.50 × 10^−2^	1.29 × 10^−2^	1.78 × 10^−2^	1.33 × 10^−2^
	Rank	5	4	1	3	2
F6	Min	1.37 × 10^6^	1.61 × 10^6^	4.04 × 10^5^	3.17 × 10^4^	4.15 × 10^3^
	Mean	1.95 × 10^6^	2.03 × 10^6^	8.75 × 10^5^	6.38 × 10^5^	3.56 × 10^5^
	Std	1.18 × 10^6^	9.93 × 10^5^	1.34 × 10^6^	1.53 × 10^6^	1.01 × 10^6^
	Rank	4	5	3	2	1
F7	Min	1.92 × 10^3^	2.01 × 10^3^	2.32 × 10^3^	1.67 × 10^3^	1.89 × 10^3^
	Mean	2.01 × 10^3^	2.04 × 10^3^	2.57 × 10^3^	1.72 × 10^3^	2.06 × 10^3^
	Std	1.66 × 10^2^	9.66 × 10^1^	1.75 × 10^2^	1.11 × 10^2^	1.04 × 10^2^
	Rank	2	3	5	1	4
F8	Min	8.75 × 10^2^	8.45 × 10^2^	8.59 × 10^2^	8.27 × 10^2^	8.36 × 10^2^
	Mean	8.83 × 10^2^	8.53 × 10^2^	8.72 × 10^2^	8.35 × 10^2^	8.45 × 10^2^
	Std	1.99 × 10^1^	1.51 × 10^1^	2.23 × 10^1^	1.84 × 10^1^	1.23 × 10^1^
	Rank	5	3	4	1	2
F9	Min	1.23 × 10^4^	1.28 × 10^4^	1.02 × 10^4^	1.25 × 10^4^	1.02 × 10^4^
	Mean	1.30 × 10^4^	1.30 × 10^4^	1.15 × 10^4^	1.32 × 10^4^	1.22 × 10^4^
	Std	1.14 × 10^3^	4.99 × 10^2^	1.25 × 10^3^	7.61 × 10^2^	1.01 × 10^3^
	Rank	4	3	1	5	2
F10	Min	1.78 × 10^8^	4.88 × 10^8^	2.69 × 10^6^	2.15 × 10^5^	9.59 × 10^4^
	Mean	1.42 × 10^9^	1.27 × 10^9^	2.99 × 10^8^	5.54 × 10^8^	5.52 × 10^8^
	Std	5.64 × 10^9^	2.55 × 10^9^	2.26 × 10^9^	3.38 × 10^9^	3.75 × 10^9^
	Rank	5	4	1	3	2
F11	Min	9.22 × 10^8^	6.03 × 10^8^	4.27 × 10^8^	2.37 × 10^8^	4.92 × 10^7^
	Mean	2.74 × 10^9^	2.28 × 10^9^	1.58 × 10^9^	1.63 × 10^9^	6.01 × 10^8^
	Std	6.16 × 10^9^	5.05 × 10^9^	5.00 × 10^9^	5.63 × 10^9^	2.79 × 10^9^
	Rank	5	4	2	3	1
F12	Min	3.43 × 10^9^	1.13 × 10^9^	4.14 × 10^8^	8.25 × 10^7^	1.11 × 10^7^
	Mean	1.36 × 10^10^	4.76 × 10^9^	5.12 × 10^9^	5.34 × 10^9^	6.02 × 10^9^
	Std	2.69 × 10^10^	1.25 × 10^10^	1.86 × 10^10^	1.87 × 10^10^	2.66 × 10^10^
	Rank	5	1	2	3	4
F13	Min	8.63 × 10^6^	1.04 × 10^7^	1.58 × 10^6^	2.59 × 10^6^	1.40 × 10^6^
	Mean	1.88 × 10^7^	1.47 × 10^7^	1.07 × 10^7^	2.66 × 10^7^	7.51 × 10^6^
	Std	8.12 × 10^7^	1.45 × 10^7^	1.32 × 10^8^	1.76 × 10^8^	3.60 × 10^7^
	Rank	4	3	2	5	1
F14	Min	7.39 × 10^8^	1.92 × 10^8^	2.13 × 10^7^	9.97 × 10^6^	3.27 × 10^6^
	Mean	2.75 × 10^9^	1.57 × 10^9^	6.63 × 10^8^	1.33 × 10^9^	6.18 × 10^8^
	Std	5.66 × 10^9^	5.32 × 10^9^	2.52 × 10^9^	5.65 × 10^9^	3.78 × 10^9^
	Rank	5	4	2	3	1
F15	Min	1.29 × 10^8^	1.14 × 10^8^	1.75 × 10^6^	3.45 × 10^5^	7.50 × 10^4^
	Mean	2.88 × 10^9^	1.56 × 10^9^	4.60 × 10^8^	1.25 × 10^9^	9.35 × 10^8^
	Std	1.16 × 10^10^	5.33 × 10^9^	4.36 × 10^9^	8.99 × 10^9^	7.32 × 10^9^
	Rank	5	4	1	3	2
F16	Min	1.08 × 10^10^	3.29 × 10^8^	4.27 × 10^4^	9.93 × 10^4^	3.13 × 10^4^
	Mean	1.38 × 10^13^	5.73 × 10^12^	3.21 × 10^12^	3.68 × 10^12^	6.78 × 10^12^
	Std	9.93 × 10^13^	5.07 × 10^13^	4.32 × 10^13^	4.73 × 10^13^	9.61 × 10^13^
	Rank	5	3	1	2	4
F17	Min	2.58 × 10^7^	1.47 × 10^7^	5.26 × 10^6^	4.36 × 10^6^	3.22 × 10^6^
	Mean	4.99 × 10^7^	3.11 × 10^7^	1.77 × 10^7^	1.49 × 10^7^	1.26 × 10^7^
	Std	2.15 × 10^8^	1.07 × 10^8^	3.20 × 10^7^	7.09 × 10^7^	9.17 × 10^7^
	Rank	5	4	3	2	1
F18	Min	9.29 × 10^10^	9.32 × 10^10^	4.50 × 10^9^	1.00 × 10^6^	2.96 × 10^5^
	Mean	1.08 × 10^14^	3.06 × 10^14^	2.44 × 10^14^	1.18 × 10^15^	7.94 × 10^13^
	Std	1.14 × 10^15^	3.61 × 10^15^	3.61 × 10^15^	1.30 × 10^16^	1.16 × 10^15^
	Rank	2	4	3	5	1
F19	Min	9.43 × 10^3^	8.26 × 10^3^	6.87 × 10^3^	5.55 × 10^3^	5.34 × 10^3^
	Mean	1.03 × 10^4^	9.07 × 10^3^	8.34 × 10^3^	7.38 × 10^3^	7.18 × 10^3^
	Std	2.11 × 10^3^	1.39 × 10^3^	1.42 × 10^3^	2.12 × 10^3^	2.48 × 10^3^
	Rank	5	4	3	2	1
F20	Min	6.39 × 10^3^	6.28 × 10^3^	2.28 × 10^4^	6.48 × 10^3^	3.89 × 10^3^
	Mean	1.82 × 10^4^	1.14 × 10^4^	3.06 × 10^4^	1.79 × 10^4^	1.25 × 10^4^
	Std	2.95 × 10^4^	1.84 × 10^4^	2.50 × 10^4^	2.81 × 10^4^	2.64 × 10^4^
	Rank	4	1	5	3	2
F21	Min	1.39 × 10^4^	1.38 × 10^4^	3.11 × 10^3^	1.25 × 10^4^	5.01 × 10^3^
	Mean	1.43 × 10^4^	1.42 × 10^4^	3.47 × 10^3^	1.39 × 10^4^	5.97 × 10^3^
	Std	8.58 × 10^2^	7.22 × 10^2^	1.34 × 10^3^	1.28 × 10^3^	2.05 × 10^3^
	Rank	5	4	1	3	2
F22	Min	3.98 × 10^4^	2.75 × 10^4^	1.85 × 10^4^	6.50 × 10^3^	3.37 × 10^3^
	Mean	5.08 × 10^4^	3.66 × 10^4^	2.77 × 10^4^	2.64 × 10^4^	1.26 × 10^4^
	Std	2.05 × 10^4^	1.89 × 10^4^	2.10 × 10^4^	3.48 × 10^4^	2.23 × 10^4^
	Rank	5	4	3	2	1
F23	Min	3.45 × 10^4^	2.14 × 10^4^	1.79 × 10^4^	3.95 × 10^3^	3.60 × 10^3^
	Mean	4.20 × 10^4^	3.06 × 10^4^	2.50 × 10^4^	1.20 × 10^4^	7.71 × 10^3^
	Std	1.39 × 10^4^	1.64 × 10^4^	1.68 × 10^4^	1.63 × 10^4^	1.19 × 10^4^
	Rank	5	4	3	2	1
F24	Min	6.27 × 10^3^	6.44 × 10^3^	4.36 × 10^3^	4.11 × 10^3^	3.64 × 10^3^
	Mean	8.40 × 10^3^	8.17 × 10^3^	6.62 × 10^3^	5.80 × 10^3^	5.39 × 10^3^
	Std	5.23 × 10^3^	4.46 × 10^3^	7.80 × 10^3^	3.49 × 10^3^	5.72 × 10^3^
	Rank	5	4	3	2	1
F25	Min	4.69 × 10^3^	1.22 × 10^4^	4.34 × 10^3^	4.97 × 10^3^	4.28 × 10^3^
	Mean	6.48 × 10^3^	1.59 × 10^4^	5.82 × 10^3^	6.58 × 10^3^	5.56 × 10^3^
	Std	8.42 × 10^3^	9.54 × 10^3^	5.49 × 10^3^	6.68 × 10^3^	4.44 × 10^3^
	Rank	3	5	2	4	1
F26	Min	4.95 × 10^3^	4.74 × 10^3^	3.62 × 10^3^	3.69 × 10^3^	4.26 × 10^3^
	Mean	5.12 × 10^3^	4.91 × 10^3^	3.84 × 10^3^	3.90 × 10^3^	4.49 × 10^3^
	Std	3.24 × 10^2^	4.48 × 10^2^	3.86 × 10^2^	6.30 × 10^2^	2.84 × 10^2^
	Rank	5	4	1	2	3
F27	Min	3.87 × 10^3^	3.37 × 10^3^	3.26 × 10^3^	3.18 × 10^3^	3.19 × 10^3^
	Mean	4.53 × 10^3^	3.61 × 10^3^	3.58 × 10^3^	3.71 × 10^3^	3.51 × 10^3^
	Std	1.66 × 10^3^	8.75 × 10^2^	1.12 × 10^3^	1.40 × 10^3^	1.20 × 10^3^
	Rank	5	3	2	4	1
F28	Min	5.19 × 10^9^	2.34 × 10^10^	1.43 × 10^8^	2.01 × 10^8^	3.48 × 10^5^
	Mean	3.60 × 10^12^	8.17 × 10^12^	2.94 × 10^13^	2.44 × 10^13^	1.85 × 10^13^
	Std	6.27 × 10^13^	3.59 × 10^13^	3.58 × 10^14^	2.80 × 10^14^	3.03 × 10^14^
	Rank	1	2	5	4	3
F29	Min	2.59 × 10^10^	2.40 × 10^10^	7.99 × 10^9^	3.44 × 10^9^	4.81 × 10^7^
	Mean	1.24 × 10^13^	2.82 × 10^13^	5.22 × 10^12^	8.45 × 10^12^	4.12 × 10^12^
	Std	8.85 × 10^13^	3.49 × 10^14^	7.82 × 10^13^	9.46 × 10^13^	8.00 × 10^13^
	Rank	4	5	2	3	1
Average Rank		4.379	3.586	2.552	2.759	1.724
Combined Rank		5	4	2	3	1

**Table 4:** CEC2017 test results: 30 dimensions. Bold text indicates the optimal values.

Dim = 30										
Func.	Index	ImWOA	CSA	HHO	EHO	BES	WOA	ImWOA1	ImWOA2	ImWOA3
F1	Min	4.43 × 10^6^	6.63 × 10^10^	4.59 × 10^9^	4.04 × 10^10^	5.67 × 10^10^	1.87 × 10^10^	4.53 × 10^10^	2.44 × 10^10^	7.75 × 10^9^
	Mean	**2.81 × 10^9^**	7.81 × 10^10^	2.35 × 10^10^	4.64 × 10^10^	5.68 × 10^10^	2.94 × 10^10^	5.95 × 10^10^	3.17 × 10^10^	1.21 × 10^10^
	Std	1.15 × 10^10^	1.06 × 10^10^	1.63 × 10^10^	5.50 × 10^9^	4.80 × 10^8^	1.15 × 10^10^	2.50 × 10^10^	1.22 × 10^10^	1.05 × 10^10^
	Rank	1	9	3	6	7	4	8	5	2
F2	Min	2.13 × 10^2^	1.05 × 10^5^	2.76 × 10^4^	7.90 × 10^4^	1.01 × 10^5^	5.28 × 10^4^	8.00 × 10^4^	8.00 × 10^4^	4.67 × 10^4^
	Mean	**8.51 × 10^3^**	1.26 × 10^5^	6.61 × 10^4^	8.92 × 10^4^	1.02 × 10^5^	7.38 × 10^4^	1.14 × 10^5^	8.60 × 10^4^	5.39 × 10^4^
	Std	2.01 × 10^4^	2.11 × 10^4^	3.24 × 10^4^	1.14 × 10^4^	4.44 × 10^3^	2.57 × 10^4^	6.01 × 10^4^	1.39 × 10^4^	1.61 × 10^4^
	Rank	1	9	3	6	7	4	8	5	2
F3	Min	3.47 × 10^2^	1.74 × 10^4^	1.10 × 10^3^	8.44 × 10^3^	1.44 × 10^4^	2.37 × 10^3^	8.85 × 10^3^	3.40 × 10^3^	1.74 × 10^3^
	Mean	**7.31 × 10^2^**	2.27 × 10^4^	5.51 × 10^3^	1.06 × 10^4^	1.45 × 10^4^	5.41 × 10^3^	1.35 × 10^4^	5.02 × 10^3^	2.33 × 10^3^
	Std	1.82 × 10^3^	5.53 × 10^3^	5.17 × 10^3^	2.56 × 10^3^	3.28 × 10^2^	4.49 × 10^3^	1.10 × 10^4^	6.04 × 10^3^	1.42 × 10^3^
	Rank	1	9	5	6	8	4	7	3	2
F4	Min	9.06 × 10^2^	7.89 × 10^4^	9.86 × 10^3^	5.55 × 10^4^	7.27 × 10^4^	3.20 × 10^4^	5.34 × 10^4^	3.83 × 10^4^	2.16 × 10^4^
	Mean	**5.12 × 10^3^**	9.48 × 10^4^	3.48 × 10^4^	6.41 × 10^4^	7.28 × 10^4^	4.45 × 10^4^	7.63 × 10^4^	4.40 × 10^4^	2.97 × 10^4^
	Std	1.62 × 10^4^	1.35 × 10^4^	2.00 × 10^4^	8.64 × 10^3^	9.59 × 10^2^	1.26 × 10^4^	2.59 × 10^4^	1.29 × 10^4^	1.69 × 10^4^
	Rank	1	9	3	6	7	5	8	4	2
F5	Min	5.00 × 10^2^	5.00 × 10^2^	5.00 × 10^2^	5.00 × 10^2^	5.00 × 10^2^	5.00 × 10^2^	5.00 × 10^2^	5.00 × 10^2^	5.00 × 10^2^
	Mean	**5.00 × 10^2^**	5.00 × 10^2^	5.00 × 10^2^	5.00 × 10^2^	5.00 × 10^2^	5.00 × 10^2^	5.00 × 10^2^	5.00 × 10^2^	5.00 × 10^2^
	Std	5.10 × 10^−3^	1.10 × 10^−2^	1.40 × 10^−2^	6.50 × 10^−3^	2.39 × 10^−3^	1.29 × 10^−2^	2.89 × 10^−2^	1.49 × 10^−2^	9.69 × 10^−3^
	Rank	1	9	3	5	6	2	8	4	7
F6	Min	1.13 × 10^3^	2.59 × 10^6^	3.02 × 10^5^	1.80 × 10^6^	2.61 × 10^6^	8.40 × 10^5^	1.42 × 10^6^	1.79 × 10^6^	6.76 × 10^5^
	Mean	**1.21 × 10^5^**	3.09 × 10^6^	1.18 × 10^6^	2.09 × 10^6^	2.61 × 10^6^	1.27 × 10^6^	2.54 × 10^6^	1.91 × 10^6^	8.85 × 10^5^
	Std	4.47 × 10^5^	3.47 × 10^5^	7.54 × 10^5^	2.98 × 10^5^	2.48 × 10^4^	4.66 × 10^5^	8.45 × 10^5^	3.73 × 10^5^	5.58 × 10^5^
	Rank	1	9	3	6	8	4	7	5	2
F7	Min	1.24 × 10^3^	1.53 × 10^3^	1.27 × 10^3^	1.34 × 10^3^	1.35 × 10^3^	1.50 × 10^3^	1.28 × 10^3^	1.39 × 10^3^	1.38 × 10^3^
	Mean	**1.32 × 10^3^**	1.61 × 10^3^	1.34 × 10^3^	1.37 × 10^3^	1.35 × 10^3^	1.52 × 10^3^	1.48 × 10^3^	1.41 × 10^3^	1.45 × 10^3^
	Std	5.85 × 10^1^	7.33 × 10^1^	6.64 × 10^1^	3.84 × 10^1^	1.13 × 10^1^	3.50 × 10^1^	2.52 × 10^2^	4.10 × 10^1^	1.29 × 10^2^
	Rank	1	9	2	4	3	8	7	5	6
F8	Min	8.07 × 10^2^	8.53 × 10^2^	8.14 × 10^2^	8.26 × 10^2^	8.34 × 10^2^	8.26 × 10^2^	8.29 × 10^2^	8.18 × 10^2^	8.29 × 10^2^
	Mean	**8.09 × 10^2^**	8.61 × 10^2^	8.22 × 10^2^	8.30 × 10^2^	8.34 × 10^2^	8.32 × 10^2^	8.54 × 10^2^	8.22 × 10^2^	8.31 × 10^2^
	Std	6.27 × 10^0^	8.53 × 10^0^	8.82 × 10^0^	3.69 × 10^0^	9.20 × 10^−1^	7.81 × 10^0^	3.34 × 10^1^	1.15 × 10^1^	5.42 × 10^0^
	Rank	1	9	2	4	7	6	8	3	5
F9	Min	4.88 × 10^3^	8.84 × 10^3^	5.66 × 10^3^	8.51 × 10^3^	8.23 × 10^3^	6.75 × 10^3^	7.60 × 10^3^	6.80 × 10^3^	7.22 × 10^3^
	Mean	**5.82 × 10^3^**	9.18 × 10^3^	6.90 × 10^3^	8.75 × 10^3^	8.25 × 10^3^	7.25 × 10^3^	8.62 × 10^3^	7.26 × 10^3^	7.37 × 10^3^
	Std	8.69 × 10^2^	3.11 × 10^2^	9.32 × 10^2^	2.66 × 10^2^	1.47 × 10^2^	5.76 × 10^2^	8.35 × 10^2^	6.96 × 10^2^	3.73 × 10^2^
	Rank	1	9	2	8	6	3	7	4	5
F10	Min	1.19 × 10^5^	2.16 × 10^9^	1.42 × 10^6^	6.00 × 10^8^	1.78 × 10^9^	9.43 × 10^6^	1.04 × 10^9^	9.41 × 10^6^	2.75 × 10^6^
	Mean	1.10 × 10^8^	3.75 × 10^9^	3.25 × 10^8^	1.17 × 10^9^	1.79 × 10^9^	3.30 × 10^8^	2.87 × 10^9^	1.86 × 10^8^	**5.83 × 10^7^**
	Std	9.10 × 10^8^	2.31 × 10^9^	9.96 × 10^8^	1.08 × 10^9^	1.84 × 10^8^	1.22 × 10^9^	2.86 × 10^9^	9.63 × 10^8^	8.93 × 10^8^
	Rank	2	9	4	6	7	5	8	3	1
F11	Min	7.54 × 10^6^	9.58 × 10^9^	1.62 × 10^8^	5.87 × 10^9^	1.13 × 10^10^	2.85 × 10^8^	1.06 × 10^10^	1.84 × 10^9^	2.21 × 10^8^
	Mean	**2.70 × 10^8^**	1.23 × 10^10^	2.87 × 10^9^	7.97 × 10^9^	1.13 × 10^10^	1.75 × 10^9^	1.25 × 10^10^	2.16 × 10^9^	6.80 × 10^8^
	Std	1.61 × 10^9^	2.80 × 10^9^	3.81 × 10^9^	2.65 × 10^9^	3.63 × 10^8^	2.45 × 10^9^	3.24 × 10^9^	9.53 × 10^8^	1.46 × 10^9^
	Rank	1	8	5	6	7	3	9	4	2
F12	Min	4.32 × 10^5^	1.12 × 10^10^	8.93 × 10^7^	5.88 × 10^9^	1.24 × 10^10^	7.14 × 10^8^	1.40 × 10^10^	1.92 × 10^9^	2.85 × 10^8^
	Mean	**2.48 × 10^8^**	1.47 × 10^10^	3.63 × 10^9^	8.00 × 10^9^	1.25 × 10^10^	2.67 × 10^9^	1.59 × 10^10^	3.19 × 10^9^	1.11 × 10^9^
	Std	1.80 × 10^9^	3.92 × 10^9^	4.85 × 10^9^	2.86 × 10^9^	2.76 × 10^8^	3.05 × 10^9^	3.86 × 10^9^	4.12 × 10^9^	3.39 × 10^9^
	Rank	1	8	5	6	7	3	9	4	2
F13	Min	1.98 × 10^5^	9.74 × 10^6^	3.16 × 10^6^	6.74 × 10^6^	7.21 × 10^6^	2.23 × 10^6^	3.56 × 10^6^	4.23 × 10^6^	1.85 × 10^6^
	Mean	**7.68 × 10^5^**	2.00 × 10^7^	7.52 × 10^6^	9.43 × 10^6^	7.42 × 10^6^	5.34 × 10^6^	8.69 × 10^6^	9.01 × 10^6^	4.22 × 10^6^
	Std	3.05 × 10^6^	1.84 × 10^7^	9.00 × 10^6^	5.01 × 10^6^	2.38 × 10^6^	9.20 × 10^6^	1.13 × 10^7^	2.29 × 10^7^	1.08 × 10^7^
	Rank	1	9	5	8	4	3	6	7	2
F14	Min	5.48 × 10^5^	5.13 × 10^9^	3.10 × 10^7^	2.32 × 10^9^	6.23 × 10^9^	8.12 × 10^7^	3.50 × 10^9^	1.20 × 10^9^	6.30 × 10^7^
	Mean	**1.05 × 10^8^**	7.21 × 10^9^	1.45 × 10^9^	3.62 × 10^9^	6.25 × 10^9^	8.93 × 10^8^	4.78 × 10^9^	1.96 × 10^9^	5.51 × 10^8^
	Std	8.58 × 10^8^	2.32 × 10^9^	2.29 × 10^9^	1.51 × 10^9^	2.20 × 10^8^	1.79 × 10^9^	1.84 × 10^9^	2.24 × 10^9^	2.14 × 10^9^
	Rank	1	9	4	6	8	3	7	5	2
F15	Min	6.99 × 10^3^	1.13 × 10^9^	1.24 × 10^6^	2.09 × 10^8^	9.68 × 10^8^	4.67 × 10^6^	9.70 × 10^7^	7.49 × 10^7^	1.05 × 10^8^
	Mean	**3.31 × 10^7^**	2.70 × 10^9^	5.19 × 10^8^	7.44 × 10^8^	9.87 × 10^8^	2.76 × 10^8^	1.05 × 10^9^	7.27 × 10^8^	1.77 × 10^8^
	Std	4.79 × 10^8^	2.99 × 10^9^	1.30 × 10^9^	1.34 × 10^9^	2.26 × 10^8^	1.26 × 10^9^	2.89 × 10^9^	3.07 × 10^9^	2.68 × 10^8^
	Rank	1	9	4	6	7	3	8	5	2
F16	Min	4.89 × 10^3^	1.06 × 10^13^	4.13 × 10^5^	1.10 × 10^12^	1.62 × 10^13^	1.42 × 10^7^	1.71 × 10^11^	9.20 × 10^7^	1.01 × 10^5^
	Mean	**1.12 × 10^11^**	2.66 × 10^14^	2.42 × 10^14^	1.27 × 10^14^	2.05 × 10^13^	4.12 × 10^13^	1.38 × 10^13^	1.28 × 10^14^	2.11 × 10^12^
	Std	2.46 × 10^12^	1.02 × 10^15^	8.88 × 10^14^	9.59 × 10^14^	7.40 × 10^13^	4.23 × 10^14^	5.65 × 10^13^	1.73 × 10^15^	1.98 × 10^13^
	Rank	1	9	8	6	4	5	3	7	2
F17	Min	5.78 × 10^5^	2.71 × 10^7^	1.56 × 10^6^	8.95 × 10^6^	8.29 × 10^6^	4.82 × 10^5^	1.20 × 10^7^	1.81 × 10^6^	9.41 × 10^6^
	Mean	**2.28 × 10^6^**	5.50 × 10^7^	6.84 × 10^6^	1.82 × 10^7^	8.89 × 10^6^	2.52 × 10^6^	4.16 × 10^7^	3.77 × 10^6^	1.33 × 10^7^
	Std	1.69 × 10^7^	4.76 × 10^7^	1.42 × 10^7^	2.93 × 10^7^	7.47 × 10^6^	9.55 × 10^6^	4.62 × 10^7^	6.85 × 10^6^	1.42 × 10^7^
	Rank	1	9	4	7	5	2	8	3	6
F18	Min	7.98 × 10^4^	9.22 × 10^12^	1.05 × 10^10^	2.42 × 10^11^	3.51 × 10^13^	4.73 × 10^9^	1.82 × 10^12^	9.46 × 10^11^	5.20 × 10^10^
	Mean	**5.89 × 10^11^**	3.02 × 10^14^	4.38 × 10^13^	7.97 × 10^13^	3.83 × 10^13^	4.67 × 10^13^	2.30 × 10^14^	2.67 × 10^12^	5.94 × 10^12^
	Std	1.11 × 10^13^	1.44 × 10^15^	3.14 × 10^14^	6.57 × 10^14^	4.75 × 10^13^	5.05 × 10^14^	7.49 × 10^14^	7.40 × 10^12^	1.11 × 10^14^
	Rank	1	9	5	7	4	6	8	2	3
F19	Min	4.56 × 10^3^	1.34 × 10^4^	9.02 × 10^3^	7.35 × 10^3^	9.83 × 10^3^	1.15 × 10^4^	9.62 × 10^3^	9.91 × 10^3^	5.43 × 10^3^
	Mean	**6.59 × 10^3^**	1.61 × 10^4^	1.07 × 10^4^	8.43 × 10^3^	9.90 × 10^3^	1.21 × 10^4^	1.21 × 10^4^	1.03 × 10^4^	7.64 × 10^3^
	Std	2.53 × 10^3^	2.93 × 10^3^	1.62 × 10^3^	1.44 × 10^3^	4.71 × 10^2^	1.47 × 10^3^	5.19 × 10^3^	2.04 × 10^3^	4.22 × 10^3^
	Rank	1	9	6	3	4	7	8	5	2
F20	Min	4.56 × 10^3^	1.34 × 10^4^	9.02 × 10^3^	7.35 × 10^3^	9.83 × 10^3^	1.15 × 10^4^	9.62 × 10^3^	9.91 × 10^3^	5.43 × 10^3^
	Mean	**6.59 × 10^3^**	1.61 × 10^4^	1.07 × 10^4^	8.43 × 10^3^	9.90 × 10^3^	1.21 × 10^4^	1.21 × 10^4^	1.03 × 10^4^	7.64 × 10^3^
	Std	2.53 × 10^3^	2.93 × 10^3^	1.62 × 10^3^	1.44 × 10^3^	4.71 × 10^2^	1.47 × 10^3^	5.19 × 10^3^	2.04 × 10^3^	4.22 × 10^3^
	Rank	1	9	6	3	4	7	8	5	2
F21	Min	2.32 × 10^3^	4.40 × 10^3^	4.38 × 10^3^	3.45 × 10^3^	4.69 × 10^3^	4.13 × 10^3^	3.12 × 10^3^	2.89 × 10^3^	2.85 × 10^3^
	Mean	**2.41 × 10^3^**	5.07 × 10^3^	4.95 × 10^3^	4.01 × 10^3^	4.70 × 10^3^	4.41 × 10^3^	3.32 × 10^3^	3.60 × 10^3^	2.88 × 10^3^
	Std	4.50 × 10^2^	7.66 × 10^2^	4.77 × 10^2^	6.57 × 10^2^	8.91 × 10^1^	4.23 × 10^2^	6.52 × 10^2^	1.29 × 10^3^	1.39 × 10^2^
	Rank	1	9	8	5	7	6	3	4	2
F22	Min	2.71 × 10^3^	6.55 × 10^4^	1.55 × 10^4^	5.27 × 10^4^	5.89 × 10^4^	3.33 × 10^4^	5.67 × 10^4^	5.42 × 10^4^	1.79 × 10^4^
	Mean	**5.46 × 10^3^**	7.29 × 10^4^	3.69 × 10^4^	5.76 × 10^4^	5.89 × 10^4^	4.28 × 10^4^	6.97 × 10^4^	5.79 × 10^4^	2.31 × 10^4^
	Std	7.36 × 10^3^	7.87 × 10^3^	1.51 × 10^4^	4.07 × 10^3^	4.24 × 10^2^	9.25 × 10^3^	1.83 × 10^4^	8.09 × 10^3^	1.07 × 10^4^
	Rank	1	9	3	5	7	4	8	6	2
F23	Min	2.63 × 10^3^	3.64 × 10^4^	7.97 × 10^3^	3.06 × 10^4^	3.35 × 10^4^	2.33 × 10^4^	3.35 × 10^4^	2.46 × 10^4^	1.53 × 10^4^
	Mean	**5.01 × 10^3^**	4.40 × 10^4^	1.96 × 10^4^	3.26 × 10^4^	3.35 × 10^4^	2.75 × 10^4^	4.27 × 10^4^	2.70 × 10^4^	1.79 × 10^4^
	Std	7.03 × 10^3^	6.26 × 10^3^	8.73 × 10^3^	1.28 × 10^3^	1.37 × 10^2^	4.60 × 10^3^	1.17 × 10^4^	5.23 × 10^3^	6.54 × 10^3^
	Rank	1	9	3	6	7	5	8	4	2
F24	Min	2.86 × 10^3^	8.89 × 10^3^	3.28 × 10^3^	5.61 × 10^3^	7.29 × 10^3^	3.78 × 10^3^	6.05 × 10^3^	4.43 × 10^3^	3.47 × 10^3^
	Mean	**2.99 × 10^3^**	1.11 × 10^4^	4.51 × 10^3^	6.16 × 10^3^	7.30 × 10^3^	4.67 × 10^3^	8.96 × 10^3^	4.71 × 10^3^	3.81 × 10^3^
	Std	5.85 × 10^2^	2.44 × 10^3^	1.53 × 10^3^	6.15 × 10^2^	8.85 × 10^1^	1.43 × 10^3^	5.62 × 10^3^	1.39 × 10^3^	1.66 × 10^3^
	Rank	1	9	3	6	7	4	8	5	2
F25	Min	3.30 × 10^3^	1.06 × 10^4^	4.79 × 10^3^	9.34 × 10^3^	7.97 × 10^3^	4.75 × 10^3^	4.11 × 10^3^	3.57 × 10^3^	4.60 × 10^3^
	Mean	**3.86 × 10^3^**	1.29 × 10^4^	6.87 × 10^3^	1.17 × 10^4^	8.04 × 10^3^	5.51 × 10^3^	4.12 × 10^3^	4.02 × 10^3^	4.86 × 10^3^
	Std	2.85 × 10^3^	3.61 × 10^3^	2.69 × 10^3^	2.80 × 10^3^	5.78 × 10^2^	1.79 × 10^3^	1.03 × 10^2^	1.24 × 10^3^	2.28 × 10^3^
	Rank	1	9	6	8	7	5	3	2	4
F26	Min	3.52 × 10^3^	4.28 × 10^3^	3.81 × 10^3^	4.34 × 10^3^	3.81 × 10^3^	3.97 × 10^3^	3.55 × 10^3^	3.85 × 10^3^	3.72 × 10^3^
	Mean	3.63 × 10^3^	4.49 × 10^3^	3.97 × 10^3^	4.58 × 10^3^	3.81 × 10^3^	4.01 × 10^3^	**3.58 × 10^3^**	3.91 × 10^3^	3.74 × 10^3^
	Std	1.38 × 10^2^	2.29 × 10^2^	1.32 × 10^2^	2.69 × 10^2^	5.92 × 10^1^	1.00 × 10^2^	2.03 × 10^2^	1.08 × 10^2^	9.24 × 10^1^
	Rank	2	8	6	9	4	7	1	5	3
F27	Min	3.12 × 10^3^	6.61 × 10^3^	3.52 × 10^3^	5.34 × 10^3^	6.13 × 10^3^	3.71 × 10^3^	5.06 × 10^3^	4.32 × 10^3^	3.46 × 10^3^
	Mean	**3.26 × 10^3^**	7.55 × 10^3^	4.59 × 10^3^	6.04 × 10^3^	6.15 × 10^3^	4.14 × 10^3^	5.40 × 10^3^	4.54 × 10^3^	3.76 × 10^3^
	Std	3.58 × 10^2^	9.60 × 10^2^	9.91 × 10^2^	1.01 × 10^3^	1.72 × 10^2^	5.43 × 10^2^	1.10 × 10^3^	6.63 × 10^2^	6.52 × 10^2^
	Rank	1	9	5	7	8	3	6	4	2
F28	Min	4.63 × 10^4^	1.08 × 10^12^	1.82 × 10^9^	9.96 × 10^10^	3.55 × 10^13^	5.64 × 10^8^	9.46 × 10^10^	1.55 × 10^10^	1.26 × 10^9^
	Mean	1.20 × 10^12^	3.27 × 10^13^	3.95 × 10^12^	2.94 × 10^12^	3.61 × 10^13^	2.27 × 10^12^	6.73 × 10^13^	1.74 × 10^13^	**9.65 × 10^10^**
	Std	2.55 × 10^13^	1.51 × 10^14^	4.05 × 10^13^	2.31 × 10^13^	8.72 × 10^12^	2.63 × 10^13^	4.67 × 10^14^	1.41 × 10^14^	1.22 × 10^12^
	Rank	2	7	5	4	8	3	9	6	1
F29	Min	1.24 × 10^6^	2.92 × 10^11^	1.26 × 10^9^	1.29 × 10^10^	2.85 × 10^11^	2.59 × 10^9^	5.55 × 10^10^	1.58 × 10^10^	2.60 × 10^8^
	Mean	5.20 × 10^10^	5.79 × 10^12^	7.18 × 10^11^	5.58 × 10^11^	3.15 × 10^11^	5.93 × 10^11^	2.24 × 10^12^	4.66 × 10^11^	**3.10 × 10^10^**
	Std	7.33 × 10^11^	2.72 × 10^13^	5.19 × 10^12^	4.48 × 10^12^	5.28 × 10^11^	5.62 × 10^12^	3.61 × 10^13^	6.77 × 10^12^	2.29 × 10^11^
	Rank	2	9	7	5	3	6	8	4	1
Total		**25**	0	0	0	0	0	1	0	3

**Table 5:** CEC2017 test results: 100 dimensions. Bold text indicates the optimal values.

Dim = 100										
Func.	Index	ImWOA	CSA	HHO	EHO	BES	WOA	ImWOA1	ImWOA2	ImWOA3
F1	Min	5.84 × 10^8^	3.79 × 10^11^	1.13 × 10^11^	2.64 × 10^11^	2.73 × 10^11^	1.88 × 10^11^	2.49 × 10^11^	1.97 × 10^11^	1.48 × 10^11^
	Mean	**3.62 × 10^10^**	4.32 × 10^11^	1.87 × 10^11^	2.66 × 10^11^	2.73 × 10^11^	2.23 × 10^11^	3.20 × 10^11^	2.08 × 10^11^	1.69 × 10^11^
	Std	6.87 × 10^10^	4.28 × 10^10^	5.12 × 10^10^	3.69 × 10^9^	6.70 × 10^8^	3.89 × 10^10^	1.35 × 10^11^	2.47 × 10^10^	5.19 × 10^10^
	Rank	1	9	3	6	7	5	8	4	2
F2	Min	1.77 × 10^4^	5.01 × 10^5^	2.15 × 10^5^	3.34 × 10^5^	3.59 × 10^5^	3.14 × 10^5^	3.05 × 10^5^	3.32 × 10^5^	3.35 × 10^5^
	Mean	**1.46 × 10^5^**	5.75 × 10^5^	2.99 × 10^5^	3.60 × 10^5^	3.60 × 10^5^	4.20 × 10^5^	4.09 × 10^5^	3.45 × 10^5^	4.06 × 10^5^
	Std	1.60 × 10^5^	6.64 × 10^4^	7.63 × 10^4^	3.35 × 10^4^	1.35 × 10^4^	1.33 × 10^5^	1.89 × 10^5^	6.94 × 10^4^	8.14 × 10^4^
	Rank	1	9	2	4	5	8	7	3	6
F3	Min	1.18 × 10^3^	1.61 × 10^5^	2.55 × 10^4^	9.67 × 10^4^	1.27 × 10^5^	4.49 × 10^4^	8.55 × 10^4^	5.63 × 10^4^	2.51 × 10^4^
	Mean	**8.35 × 10^3^**	1.90 × 10^5^	6.51 × 10^4^	1.11 × 10^5^	1.27 × 10^5^	6.92 × 10^4^	1.42 × 10^5^	6.67 × 10^4^	3.75 × 10^4^
	Std	2.15 × 10^4^	3.45 × 10^4^	3.47 × 10^4^	1.24 × 10^4^	1.09 × 10^3^	2.88 × 10^4^	9.41 × 10^4^	2.12 × 10^4^	3.87 × 10^4^
	Rank	1	9	3	6	7	5	8	4	2
F4	Min	5.81 × 10^3^	4.21 × 10^5^	1.41 × 10^5^	3.21 × 10^5^	3.41 × 10^5^	2.41 × 10^5^	3.23 × 10^5^	2.38 × 10^5^	2.02 × 10^5^
	Mean	**4.24 × 10^4^**	4.72 × 10^5^	2.33 × 10^5^	3.35 × 10^5^	3.41 × 10^5^	2.74 × 10^5^	3.95 × 10^5^	2.45 × 10^5^	2.21 × 10^5^
	Std	7.67 × 10^4^	4.68 × 10^4^	6.25 × 10^4^	8.24 × 10^3^	9.09 × 10^2^	3.98 × 10^4^	1.38 × 10^5^	2.10 × 10^4^	5.09 × 10^4^
	Rank	1	9	3	6	7	5	8	4	2
F5	Min	5.00 × 10^2^	5.00 × 10^2^	5.00 × 10^2^	5.00 × 10^2^	5.00 × 10^2^	5.00 × 10^2^	5.00 × 10^2^	5.00 × 10^2^	5.00 × 10^2^
	Mean	5.00 × 10^2^	5.00 × 10^2^	5.00 × 10^2^	5.00 × 10^2^	5.00 × 10^2^	5.00 × 10^2^	5.00 × 10^2^	5.00 × 10^2^	**5.00 × 10^2^**
	Std	2.59 × 10^−2^	1.23 × 10^−2^	1.94 × 10^−2^	4.79 × 10^−3^	1.39 × 10^−3^	2.50 × 10^−2^	5.52 × 10^−2^	1.25 × 10^−2^	1.61 × 10^−2^
	Rank	4	9	2	6	7	5	8	3	1
F6	Min	3.35 × 10^4^	1.47 × 10^7^	4.58 × 10^6^	1.03 × 10^7^	1.07 × 10^7^	7.49 × 10^6^	9.68 × 10^6^	8.79 × 10^6^	6.12 × 10^6^
	Mean	**1.41 × 10^6^**	1.61 × 10^7^	7.42 × 10^6^	1.06 × 10^7^	1.07 × 10^7^	8.69 × 10^6^	1.21 × 10^7^	9.10 × 10^6^	6.81 × 10^6^
	Std	2.65 × 10^6^	1.24 × 10^6^	1.94 × 10^6^	1.99 × 10^5^	2.52 × 10^4^	1.35 × 10^6^	4.22 × 10^6^	1.04 × 10^6^	1.58 × 10^6^
	Rank	1	9	3	6	7	4	8	5	2
F7	Min	3.40 × 10^3^	4.84 × 10^3^	3.44 × 10^3^	3.69 × 10^3^	3.76 × 10^3^	4.22 × 10^3^	3.93 × 10^3^	3.78 × 10^3^	3.74 × 10^3^
	Mean	**3.62 × 10^3^**	5.02 × 10^3^	3.64 × 10^3^	3.74 × 10^3^	3.77 × 10^3^	4.26 × 10^3^	4.39 × 10^3^	3.85 × 10^3^	3.77 × 10^3^
	Std	2.01 × 10^2^	1.95 × 10^2^	1.58 × 10^2^	7.20 × 10^1^	2.75 × 10^1^	1.45 × 10^2^	7.31 × 10^2^	1.97 × 10^2^	2.17 × 10^2^
	Rank	1	9	2	3	5	7	8	6	4
F8	Min	9.02 × 10^2^	1.22 × 10^3^	9.34 × 10^2^	1.07 × 10^3^	1.10 × 10^3^	1.03 × 10^3^	1.04 × 10^3^	1.04 × 10^3^	9.16 × 10^2^
	Mean	9.40 × 10^2^	1.26 × 10^3^	9.98 × 10^2^	1.08 × 10^3^	1.10 × 10^3^	1.09 × 10^3^	1.17 × 10^3^	1.04 × 10^3^	**9.36 × 10^2^**
	Std	4.96 × 10^1^	3.29 × 10^1^	5.29 × 10^1^	1.17 × 10^1^	3.03 × 10^0^	5.18 × 10^1^	1.30 × 10^2^	7.13 × 10^0^	6.17 × 10^1^
	Rank	2	9	3	5	7	6	8	4	1
F9	Min	2.21 × 10^4^	3.38 × 10^4^	2.79 × 10^4^	3.35 × 10^4^	3.30 × 10^4^	3.09 × 10^4^	3.43 × 10^4^	3.26 × 10^4^	3.21 × 10^4^
	Mean	**2.69 × 10^4^**	3.43 × 10^4^	3.12 × 10^4^	3.39 × 10^4^	3.31 × 10^4^	3.14 × 10^4^	3.46 × 10^4^	3.31 × 10^4^	3.25 × 10^4^
	Std	2.35 × 10^3^	6.36 × 10^2^	1.72 × 10^3^	4.39 × 10^2^	3.03 × 10^2^	8.97 × 10^2^	7.08 × 10^2^	8.80 × 10^2^	7.69 × 10^2^
	Rank	1	8	2	7	6	3	9	5	4
F10	Min	4.82 × 10^5^	9.49 × 10^10^	3.61 × 10^9^	4.23 × 10^10^	5.73 × 10^10^	1.48 × 10^10^	5.65 × 10^10^	1.85 × 10^10^	7.90 × 10^9^
	Mean	**5.31 × 10^9^**	1.21 × 10^11^	2.30 × 10^10^	5.04 × 10^10^	5.74 × 10^10^	2.79 × 10^10^	9.95 × 10^10^	2.55 × 10^10^	1.47 × 10^10^
	Std	2.46 × 10^10^	2.72 × 10^10^	2.07 × 10^10^	7.33 × 10^9^	8.06 × 10^8^	1.93 × 10^10^	6.96 × 10^10^	2.19 × 10^10^	3.18 × 10^10^
	Rank	1	9	3	6	7	5	8	4	2
F11	Min	5.55 × 10^8^	2.15 × 10^11^	4.30 × 10^10^	1.59 × 10^11^	1.90 × 10^11^	9.05 × 10^10^	1.61 × 10^11^	1.28 × 10^11^	6.33 × 10^10^
	Mean	**1.49 × 10^10^**	2.42 × 10^11^	1.09 × 10^11^	1.76 × 10^11^	1.90 × 10^11^	1.27 × 10^11^	1.89 × 10^11^	1.42 × 10^11^	8.75 × 10^10^
	Std	4.40 × 10^10^	2.40 × 10^10^	4.93 × 10^10^	1.40 × 10^10^	1.41 × 10^9^	3.33 × 10^10^	5.33 × 10^10^	3.47 × 10^10^	4.49 × 10^10^
	Rank	1	9	3	6	8	4	7	5	2
F12	Min	1.64 × 10^8^	4.13 × 10^11^	7.57 × 10^10^	2.77 × 10^11^	3.74 × 10^11^	1.50 × 10^11^	3.09 × 10^11^	1.38 × 10^11^	8.35 × 10^10^
	Mean	**2.29 × 10^10^**	4.83 × 10^11^	1.97 × 10^11^	3.11 × 10^11^	3.74 × 10^11^	2.21 × 10^11^	3.68 × 10^11^	1.72 × 10^11^	1.21 × 10^11^
	Std	8.52 × 10^10^	7.23 × 10^10^	9.63 × 10^10^	3.30 × 10^10^	3.15 × 10^9^	7.81 × 10^10^	1.28 × 10^11^	7.29 × 10^10^	9.16 × 10^10^
	Rank	1	9	4	6	8	5	7	3	2
F13	Min	4.51 × 10^6^	3.21 × 10^8^	4.11 × 10^7^	1.69 × 10^8^	2.84 × 10^8^	4.86 × 10^7^	4.44 × 10^8^	1.06 × 10^8^	4.05 × 10^7^
	Mean	**2.75 × 10^7^**	4.45 × 10^8^	1.47 × 10^8^	2.45 × 10^8^	2.87 × 10^8^	1.14 × 10^8^	4.75 × 10^8^	1.35 × 10^8^	8.16 × 10^7^
	Std	1.09 × 10^8^	1.49 × 10^8^	1.27 × 10^8^	1.06 × 10^8^	2.70 × 10^7^	1.05 × 10^8^	1.04 × 10^8^	1.10 × 10^8^	1.04 × 10^8^
	Rank	1	8	5	6	7	3	9	4	2
F14	Min	2.75 × 10^7^	7.57 × 10^10^	6.33 × 10^9^	4.65 × 10^10^	5.94 × 10^10^	2.24 × 10^10^	4.69 × 10^10^	2.59 × 10^10^	9.96 × 10^9^
	Mean	**4.89 × 10^9^**	9.20 × 10^10^	2.66 × 10^10^	5.13 × 10^10^	5.94 × 10^10^	3.46 × 10^10^	7.16 × 10^10^	3.29 × 10^10^	1.59 × 10^10^
	Std	1.93 × 10^10^	1.52 × 10^10^	1.73 × 10^10^	4.89 × 10^9^	4.46 × 10^8^	1.33 × 10^10^	3.97 × 10^10^	1.35 × 10^10^	1.36 × 10^10^
	Rank	1	9	3	6	7	5	8	4	2
F15	Min	8.77 × 10^4^	1.98 × 10^10^	1.50 × 10^8^	9.39 × 10^9^	1.70 × 10^10^	2.80 × 10^9^	1.38 × 10^10^	2.75 × 10^9^	4.19 × 10^8^
	Mean	**7.88 × 10^8^**	2.82 × 10^10^	4.57 × 10^9^	1.28 × 10^10^	1.70 × 10^10^	6.06 × 10^9^	2.35 × 10^10^	4.58 × 10^9^	1.44 × 10^9^
	Std	4.57 × 10^9^	9.38 × 10^9^	5.99 × 10^9^	2.97 × 10^9^	2.89 × 10^8^	5.08 × 10^9^	1.62 × 10^10^	6.83 × 10^9^	5.56 × 10^9^
	Rank	1	9	3	6	7	5	8	4	2
F16	Min	8.85 × 10^4^	8.17 × 10^15^	1.93 × 10^13^	2.71 × 10^15^	8.14 × 10^15^	1.61 × 10^14^	4.21 × 10^15^	3.04 × 10^15^	2.92 × 10^12^
	Mean	**3.61 × 10^13^**	1.75 × 10^16^	2.83 × 10^15^	5.63 × 10^15^	8.19 × 10^15^	2.53 × 10^15^	1.18 × 10^16^	8.74 × 10^15^	8.13 × 10^14^
	Std	3.44 × 10^14^	1.59 × 10^16^	7.86 × 10^15^	3.65 × 10^15^	6.13 × 10^14^	8.23 × 10^15^	1.01 × 10^16^	7.32 × 10^16^	5.02 × 10^15^
	Rank	1	9	4	5	6	3	8	7	2
F17	Min	1.99 × 10^7^	1.27 × 10^9^	1.74 × 10^8^	7.13 × 10^8^	1.15 × 10^9^	1.80 × 10^8^	9.18 × 10^8^	6.08 × 10^8^	1.21 × 10^8^
	Mean	**1.50 × 10^8^**	1.74 × 10^9^	7.80 × 10^8^	9.97 × 10^8^	1.16 × 10^9^	4.57 × 10^8^	1.70 × 10^9^	1.20 × 10^9^	2.71 × 10^8^
	Std	5.73 × 10^8^	5.48 × 10^8^	6.85 × 10^8^	4.28 × 10^8^	9.23 × 10^7^	4.74 × 10^8^	7.27 × 10^8^	1.28 × 10^9^	4.04 × 10^8^
	Rank	1	9	4	5	6	3	8	7	2
F18	Min	3.41 × 10^6^	5.54 × 10^15^	3.95 × 10^12^	1.01 × 10^15^	4.32 × 10^15^	1.13 × 10^14^	2.53 × 10^15^	1.81 × 10^13^	7.10 × 10^12^
	Mean	9.83 × 10^14^	1.20 × 10^16^	1.53 × 10^15^	2.40 × 10^15^	4.35 × 10^15^	1.48 × 10^15^	8.07 × 10^15^	1.45 × 10^15^	**7.59 × 10^14^**
	Std	9.05 × 10^15^	1.14 × 10^16^	4.72 × 10^15^	2.26 × 10^15^	3.10 × 10^14^	5.65 × 10^15^	1.31 × 10^16^	1.36 × 10^16^	7.61 × 10^15^
	Rank	2	9	5	6	7	4	8	3	1
F19	Min	1.20 × 10^4^	4.05 × 10^4^	2.89 × 10^4^	2.62 × 10^4^	3.41 × 10^4^	3.63 × 10^4^	3.77 × 10^4^	2.86 × 10^4^	2.39 × 10^4^
	Mean	**1.58 × 10^4^**	4.63 × 10^4^	3.35 × 10^4^	2.94 × 10^4^	3.42 × 10^4^	3.74 × 10^4^	4.33 × 10^4^	3.15 × 10^4^	2.54 × 10^4^
	Std	5.68 × 10^3^	5.63 × 10^3^	3.68 × 10^3^	3.19 × 10^3^	3.96 × 10^2^	2.50 × 10^3^	9.16 × 10^3^	5.10 × 10^3^	5.16 × 10^3^
	Rank	1	9	5	3	6	7	8	4	2
F20	Min	4.40 × 10^3^	3.96 × 10^5^	1.18 × 10^5^	2.57 × 10^5^	2.67 × 10^5^	2.08 × 10^5^	2.55 × 10^5^	1.79 × 10^5^	1.42 × 10^5^
	Mean	**3.10 × 10^4^**	4.50 × 10^5^	1.85 × 10^5^	2.59 × 10^5^	2.67 × 10^5^	2.29 × 10^5^	3.38 × 10^5^	1.90 × 10^5^	1.60 × 10^5^
	Std	6.88 × 10^4^	4.80 × 10^4^	4.45 × 10^4^	3.44 × 10^3^	5.65 × 10^2^	3.30 × 10^4^	1.42 × 10^5^	2.20 × 10^4^	4.47 × 10^4^
	Rank	1	9	3	6	7	5	8	4	2
F21	Min	1.30 × 10^4^	2.89 × 10^4^	2.06 × 10^4^	2.35 × 10^4^	2.89 × 10^4^	2.76 × 10^4^	2.80 × 10^4^	2.84 × 10^4^	2.43 × 10^4^
	Mean	**1.57 × 10^4^**	3.10 × 10^4^	2.48 × 10^4^	2.54 × 10^4^	2.89 × 10^4^	2.88 × 10^4^	2.94 × 10^4^	2.87 × 10^4^	2.49 × 10^4^
	Std	3.18 × 10^3^	1.61 × 10^3^	2.58 × 10^3^	1.62 × 10^3^	1.92 × 10^2^	1.16 × 10^3^	2.22 × 10^3^	1.12 × 10^3^	1.44 × 10^3^
	Rank	1	9	2	4	7	6	8	5	3
F22	Min	5.58 × 10^3^	2.21 × 10^5^	9.13 × 10^4^	1.26 × 10^5^	1.30 × 10^5^	1.22 × 10^5^	1.29 × 10^5^	1.15 × 10^5^	1.01 × 10^5^
	Mean	**3.08 × 10^4^**	2.41 × 10^5^	1.11 × 10^5^	1.27 × 10^5^	1.30 × 10^5^	1.27 × 10^5^	1.67 × 10^5^	1.19 × 10^5^	1.06 × 10^5^
	Std	4.02 × 10^4^	2.21 × 10^4^	1.48 × 10^4^	1.30 × 10^3^	9.62 × 10^1^	1.55 × 10^4^	7.29 × 10^4^	1.77 × 10^4^	1.50 × 10^4^
	Rank	1	9	3	5	7	6	8	4	2
F23	Min	7.85 × 10^3^	2.97 × 10^5^	1.15 × 10^5^	1.80 × 10^5^	1.89 × 10^5^	1.61 × 10^5^	1.69 × 10^5^	1.47 × 10^5^	1.39 × 10^5^
	Mean	**4.19 × 10^4^**	3.41 × 10^5^	1.48 × 10^5^	1.82 × 10^5^	1.89 × 10^5^	1.73 × 10^5^	2.27 × 10^5^	1.52 × 10^5^	1.49 × 10^5^
	Std	5.43 × 10^4^	4.19 × 10^4^	2.49 × 10^4^	2.28 × 10^3^	1.98 × 10^2^	2.70 × 10^4^	1.12 × 10^5^	1.20 × 10^4^	3.03 × 10^4^
	Rank	1	9	2	6	7	5	8	4	3
F24	Min	4.18 × 10^3^	6.92 × 10^4^	1.22 × 10^4^	4.02 × 10^4^	4.82 × 10^4^	1.83 × 10^4^	3.91 × 10^4^	2.61 × 10^4^	1.20 × 10^4^
	Mean	**8.67 × 10^3^**	9.31 × 10^4^	2.42 × 10^4^	4.34 × 10^4^	4.82 × 10^4^	2.58 × 10^4^	6.11 × 10^4^	3.02 × 10^4^	1.57 × 10^4^
	Std	1.21 × 10^4^	1.83 × 10^4^	1.15 × 10^4^	3.09 × 10^3^	3.13 × 10^2^	1.07 × 10^4^	4.30 × 10^4^	8.24 × 10^3^	1.18 × 10^4^
	Rank	1	9	3	6	7	4	8	5	2
F25	Min	9.13 × 10^3^	2.54 × 10^5^	1.11 × 10^5^	2.14 × 10^5^	1.80 × 10^5^	8.25 × 10^4^	7.65 × 10^4^	1.93 × 10^4^	9.17 × 10^4^
	Mean	**2.26 × 10^4^**	3.00 × 10^5^	1.39 × 10^5^	2.59 × 10^5^	1.81 × 10^5^	9.02 × 10^4^	8.17 × 10^4^	3.07 × 10^4^	9.88 × 10^4^
	Std	3.42 × 10^4^	4.66 × 10^4^	3.44 × 10^4^	4.78 × 10^4^	1.11 × 10^4^	2.52 × 10^4^	2.68 × 10^4^	2.92 × 10^4^	3.76 × 10^4^
	Rank	1	9	6	8	7	4	3	2	5
F26	Min	4.97 × 10^3^	1.02 × 10^4^	8.59 × 10^3^	9.96 × 10^3^	9.25 × 10^3^	9.48 × 10^3^	8.95 × 10^3^	8.18 × 10^3^	7.64 × 10^3^
	Mean	**6.08 × 10^3^**	1.08 × 10^4^	9.92 × 10^3^	1.07 × 10^4^	9.27 × 10^3^	9.70 × 10^3^	8.98 × 10^3^	8.92 × 10^3^	7.91 × 10^3^
	Std	6.96 × 10^2^	6.78 × 10^2^	8.74 × 10^2^	7.30 × 10^2^	1.74 × 10^2^	3.75 × 10^2^	2.73 × 10^2^	1.19 × 10^3^	8.31 × 10^2^
	Rank	1	9	7	8	5	6	4	3	2
F27	Min	3.36 × 10^3^	3.11 × 10^4^	9.24 × 10^3^	2.34 × 10^4^	2.87 × 10^4^	1.37 × 10^4^	1.49 × 10^4^	1.62 × 10^4^	1.06 × 10^4^
	Mean	**2.72 × 10^15^**	3.67 × 10^4^	1.65 × 10^4^	2.61 × 10^4^	2.87 × 10^4^	1.89 × 10^4^	2.78 × 10^4^	1.86 × 10^4^	1.29 × 10^4^
	Std	5.00 × 10^3^	5.29 × 10^3^	6.10 × 10^3^	2.42 × 10^3^	2.45 × 10^2^	5.24 × 10^3^	9.78 × 10^3^	4.54 × 10^3^	4.73 × 10^3^
	Rank	1	9	3	6	8	5	7	4	2
F28	Min	5.71 × 10^7^	2.27 × 10^16^	9.79 × 10^12^	1.53 × 10^15^	1.14 × 10^16^	4.18 × 10^14^	4.53 × 10^15^	3.83 × 10^14^	1.17 × 10^14^
	Mean	**8.96 × 10^14^**	5.08 × 10^16^	3.50 × 10^15^	5.10 × 10^15^	1.15 × 10^16^	4.07 × 10^15^	3.90 × 10^16^	1.55 × 10^15^	5.74 × 10^15^
	Std	1.16 × 10^16^	5.15 × 10^16^	1.81 × 10^16^	7.43 × 10^15^	1.13 × 10^15^	2.09 × 10^16^	9.15 × 10^16^	5.40 × 10^15^	7.97 × 10^16^
	Rank	1	9	3	5	7	4	8	2	6
F29	Min	2.55 × 10^8^	8.58 × 10^15^	1.78 × 10^12^	8.79 × 10^14^	3.86 × 10^15^	9.41 × 10^13^	1.12 × 10^15^	3.43 × 10^15^	1.70 × 10^13^
	Mean	**7.25 × 10^14^**	2.35 × 10^16^	1.61 × 10^15^	1.71 × 10^15^	3.89 × 10^15^	1.98 × 10^15^	2.60 × 10^16^	4.97 × 10^15^	7.99 × 10^14^
	Std	7.99 × 10^15^	2.66 × 10^16^	9.73 × 10^15^	2.39 × 10^15^	4.24 × 10^14^	1.05 × 10^16^	4.80 × 10^16^	1.99 × 10^16^	1.11 × 10^16^
	Rank	1	8	3	4	6	5	9	7	2
Total		**26**	0	0	0	0	0	0	0	3

**Table 6:** Wilcoxon rank-sum test (Dim = 30).

Func.	CSA	HHO	EHO	BES	WOA	ImWOA1	ImWOA2	ImWOA3
F1	**5.57 × 10^−160^**	**1.78 × 10^−136^**	**8.63 × 10^−154^**	**5.68 × 10^−156^**	**7.60 × 10^−145^**	**6.73 × 10^−156^**	**2.56 × 10^−146^**	**9.38 × 10^−126^**
F2	**6.08 × 10^−161^**	**2.06 × 10^−139^**	**3.54 × 10^−152^**	**5.00 × 10^−156^**	**2.32 × 10^−146^**	**3.49 × 10^−154^**	**4.85 × 10^−151^**	**2.30 × 10^−138^**
F3	**8.40 × 10^−164^**	**3.35 × 10^−146^**	**1.05 × 10^−159^**	**2.28 × 10^−162^**	**2.35 × 10^−150^**	**5.90 × 10^−160^**	**4.14 × 10^−150^**	**1.08 × 10^−138^**
F4	**4.26 × 10^−158^**	**5.36 × 10^−136^**	**9.51 × 10^−153^**	**1.01 × 10^−154^**	**6.24 × 10^−143^**	**7.63 × 10^−155^**	**1.41 × 10^−142^**	**1.54 × 10^−133^**
F5	**7.91 × 10^−164^**	**5.27 × 10^−147^**	**4.12 × 10^−160^**	**7.75 × 10^−160^**	**1.48 × 10^−148^**	**6.76 × 10^−162^**	**8.81 × 10^−157^**	**8.78 × 10^−163^**
F6	**1.22 × 10^−160^**	**2.12 × 10^−139^**	**2.13 × 10^−153^**	**8.62 × 10^−160^**	**1.55 × 10^−144^**	**1.87 × 10^−156^**	**9.41 × 10^−153^**	**1.01 × 10^−137^**
F7	**1.66 × 10^−160^**	**6.68 × 10^−03^**	**5.48 × 10^−114^**	**7.74 × 10^−113^**	**2.07 × 10^−157^**	**3.32 × 10^−09^**	**7.31 × 10^−123^**	**5.82 × 10^−128^**
F8	**9.78 × 10^−162^**	**1.26 × 10^−144^**	**6.26 × 10^−155^**	**5.52 × 10^−156^**	**2.04 × 10^−155^**	**2.92 × 10^−158^**	**2.17 × 10^−146^**	**8.13 × 10^−155^**
F9	**3.08 × 10^−159^**	**2.52 × 10^−76^**	**6.91 × 10^−155^**	**3.97 × 10^−151^**	**1.40 × 10^−115^**	**1.28 × 10^−150^**	**4.21 × 10^−113^**	**9.54 × 10^−130^**
F10	**1.48 × 10^−158^**	**2.25 × 10^−135^**	**5.99 × 10^−153^**	**5.52 × 10^−156^**	**2.04 × 10^−139^**	**1.19 × 10^−156^**	**4.20 × 10^−130^**	**2.17 × 10^−117^**
F11	**1.03 × 10^−161^**	**8.53 × 10^−143^**	**5.26 × 10^−158^**	**4.26 × 10^−161^**	**9.27 × 10^−143^**	**9.27 × 10^−162^**	**1.99 × 10^−148^**	**1.31 × 10^−129^**
F12	**2.06 × 10^−162^**	**2.23 × 10^−145^**	**1.01 × 10^−158^**	**2.29 × 10^−162^**	**2.26 × 10^−149^**	**1.64 × 10^−162^**	**7.25 × 10^−151^**	**7.70 × 10^−140^**
F13	**1.09 × 10^−154^**	**4.90 × 10^−145^**	**1.70 × 10^−148^**	**2.43 × 10^−147^**	**1.67 × 10^−142^**	**6.07 × 10^−145^**	**1.46 × 10^−142^**	**2.76 × 10^−141^**
F14	**1.83 × 10^−162^**	**1.95 × 10^−144^**	**3.45 × 10^−159^**	**2.29 × 10^−162^**	**4.16 × 10^−145^**	**1.12 × 10^−160^**	**1.03 × 10^−156^**	**4.98 × 10^−135^**
F15	**8.37 × 10^−163^**	**1.48 × 10^−150^**	**1.90 × 10^−158^**	**7.66 × 10^−160^**	**1.14 × 10^−149^**	**3.34 × 10^−156^**	**1.29 × 10^−153^**	**4.33 × 10^−154^**
F16	**3.00 × 10^−164^**	**4.97 × 10^−161^**	**7.45 × 10^−164^**	**1.14 × 10^−163^**	**4.74 × 10^−156^**	**1.42 × 10^−162^**	**8.28 × 10^−155^**	**1.06 × 10^−144^**
F17	**4.51 × 10^−162^**	**2.67 × 10^−137^**	**2.64 × 10^−157^**	**8.52 × 10^−155^**	**2.33 × 10^−04^**	**3.55 × 10^−159^**	**4.59 × 10^−124^**	**7.65 × 10^−156^**
F18	**9.95 × 10^−163^**	**3.13 × 10^−154^**	**3.43 × 10^−160^**	**1.15 × 10^−163^**	**2.67 × 10^−149^**	**9.38 × 10^−162^**	**4.39 × 10^−161^**	**2.82 × 10^−156^**
F19	**9.92 × 10^−154^**	**4.43 × 10^−131^**	**1.31 × 10^−106^**	**6.14 × 10^−124^**	**6.15 × 10^−147^**	**3.30 × 10^−131^**	**6.81 × 10^−126^**	**6.31 × 10^−08^**
F20	**9.92 × 10^−154^**	**4.43 × 10^−131^**	**1.31 × 10^−106^**	**6.14 × 10^−124^**	**6.15 × 10^−147^**	**3.30 × 10^−131^**	**6.81 × 10^−126^**	**6.31 × 10^−08^**
F21	**2.78 × 10^−161^**	**3.74 × 10^−161^**	**2.68 × 10^−159^**	**4.47 × 10^−161^**	**4.19 × 10^−161^**	**9.50 × 10^−151^**	**1.17 × 10^−149^**	**3.85 × 10^−145^**
F22	**1.09 × 10^−163^**	**4.00 × 10^−152^**	**1.37 × 10^−161^**	**2.30 × 10^−162^**	**5.08 × 10^−158^**	**1.11 × 10^−162^**	**1.79 × 10^−161^**	**4.66 × 10^−140^**
F23	**3.72 × 10^−158^**	**2.25 × 10^−138^**	**3.14 × 10^−152^**	**3.36 × 10^−152^**	**3.08 × 10^−149^**	**2.41 × 10^−156^**	**1.22 × 10^−148^**	**8.89 × 10^−140^**
F24	**7.45 × 10^−164^**	**5.55 × 10^−148^**	**1.22 × 10^−161^**	**2.28 × 10^−162^**	**1.35 × 10^−152^**	**5.45 × 10^−163^**	**2.05 × 10^−154^**	**5.12 × 10^−141^**
F25	**3.98 × 10^−152^**	**1.44 × 10^−142^**	**1.79 × 10^−151^**	**1.54 × 10^−148^**	**1.41 × 10^−137^**	**2.72 × 10^−128^**	**4.89 × 10^−103^**	**1.80 × 10^−133^**
F26	**1.73 × 10^−160^**	**3.75 × 10^−146^**	**3.36 × 10^−161^**	**5.88 × 10^−131^**	**6.58 × 10^−151^**	**8.81 × 10^−64^**	**7.02 × 10^−141^**	**4.54 × 10^−118^**
F27	**3.79 × 10^−164^**	**1.67 × 10^−152^**	**5.60 × 10^−161^**	**2.16 × 10^−162^**	**5.77 × 10^−150^**	**7.43 × 10^−158^**	**1.58 × 10^−154^**	**2.07 × 10^−139^**
F28	**9.32 × 10^−162^**	**1.52 × 10^−155^**	**4.45 × 10^−160^**	**1.17 × 10^−163^**	**1.48 × 10^−152^**	**4.42 × 10^−160^**	**2.24 × 10^−157^**	**1.63 × 10^−151^**
F29	**1.58 × 10^−160^**	**9.73 × 10^−149^**	**2.20 × 10^−155^**	**1.55 × 10^−158^**	**7.95 × 10^−150^**	**1.34 × 10^−157^**	**4.46 × 10^−154^**	**7.53 × 10^−132^**

**Table 7:** Wilcoxon rank-sum test (Dim = 100).

Func.	CSA	HHO	EHO	BES	WOA	ImWOA1	ImWOA2	ImWOA3
F1	**8.94 × 10^−162^**	**5.43 × 10^−131^**	**1.89 × 10^−153^**	**1.92 × 10^−153^**	**1.86 × 10^−145^**	**3.92 × 10^−154^**	**1.86 × 10^−142^**	**5.04 × 10^−122^**
F2	**3.53 × 10^−140^**	**1.24 × 10^−82^**	**1.32 × 10^−97^**	**2.39 × 10^−99^**	**1.51 × 10^−107^**	**1.41 × 10^−101^**	**3.21 × 10^−93^**	**3.85 × 10^−107^**
F3	**7.98 × 10^−163^**	**4.78 × 10^−146^**	**1.11 × 10^−156^**	**3.04 × 10^−157^**	**2.49 × 10^−150^**	**6.58 × 10^−157^**	**5.14 × 10^−151^**	**5.17 × 10^−130^**
F4	**3.50 × 10^−162^**	**1.57 × 10^−135^**	**6.35 × 10^−154^**	**1.06 × 10^−154^**	**8.94 × 10^−145^**	**2.03 × 10^−154^**	**8.99 × 10^−139^**	**1.86 × 10^−133^**
F5	**9.57 × 10^−158^**	**1.86 × 10^−24^**	**1.12 × 10^−41^**	**2.74 × 10^−74^**	**2.99 × 10^−17^**	**4.19 × 10^−86^**	**3.08 × 10^−3^**	**3.26 × 10^−61^**
F6	**1.41 × 10^−160^**	**1.03 × 10^−136^**	**1.77 × 10^−153^**	**1.92 × 10^−153^**	**1.33 × 10^−143^**	**1.06 × 10^−154^**	**1.72 × 10^−145^**	**1.01 × 10^−135^**
F7	**2.36 × 10^−161^**	**4.26 × 10^−9^**	**1.54 × 10^−99^**	**6.89 × 10^−102^**	**4.99 × 10^−155^**	**2.60 × 10^−141^**	**2.07 × 10^−113^**	**1.72 × 10^−102^**
F8	**1.16 × 10^−163^**	**8.95 × 10^−89^**	**1.20 × 10^−144^**	**2.32 × 10^−147^**	**6.00 × 10^−145^**	**6.32 × 10^−151^**	**1.46 × 10^−136^**	6.69 × 10^−1^
F9	**1.02 × 10^−160^**	**4.88 × 10^−120^**	**2.58 × 10^−159^**	**3.28 × 10^−156^**	**4.17 × 10^−143^**	**1.80 × 10^−161^**	**4.60 × 10^−157^**	**1.16 × 10^−155^**
F10	**9.51 × 10^−158^**	**1.28 × 10^−124^**	**1.77 × 10^−145^**	**3.06 × 10^−147^**	**1.96 × 10^−130^**	**1.67 × 10^−152^**	**9.90 × 10^−129^**	**2.12 × 10^−117^**
F11	**1.68 × 10^−158^**	**6.34 × 10^−134^**	**3.59 × 10^−149^**	**5.67 × 10^−151^**	**2.72 × 10^−140^**	**3.00 × 10^−150^**	**4.45 × 10^−145^**	**1.35 × 10^−128^**
F12	**3.81 × 10^−159^**	**2.14 × 10^−139^**	**2.13 × 10^−151^**	**3.09 × 10^−157^**	**3.00 × 10^−143^**	**2.00 × 10^−153^**	**2.14 × 10^−138^**	**7.76 × 10^−129^**
F13	**6.85 × 10^−157^**	**6.22 × 10^−139^**	**1.98 × 10^−151^**	**5.62 × 10^−156^**	**4.48 × 10^−137^**	**1.85 × 10^−157^**	**3.34 × 10^−142^**	**1.94 × 10^−121^**
F14	**3.17 × 10^−154^**	**1.55 × 10^−130^**	**3.02 × 10^−144^**	**3.15 × 10^−147^**	**2.39 × 10^−138^**	**2.37 × 10^−148^**	**1.32 × 10^−137^**	**4.47 × 10^−122^**
F15	**3.43 × 10^−159^**	**4.79 × 10^−134^**	**1.01 × 10^−152^**	**9.85 × 10^−155^**	**3.13 × 10^−142^**	**2.77 × 10^−156^**	**2.26 × 10^−140^**	**2.34 × 10^−124^**
F16	**5.84 × 10^−165^**	**9.46 × 10^−155^**	**1.47 × 10^−163^**	**5.84 × 10^−165^**	**5.95 × 10^−156^**	**1.91 × 10^−164^**	**1.75 × 10^−162^**	**3.37 × 10^−134^**
F17	**1.24 × 10^−152^**	**8.42 × 10^−136^**	**6.06 × 10^−146^**	**2.75 × 10^−147^**	**3.67 × 10^−126^**	**4.02 × 10^−151^**	**2.77 × 10^−146^**	**2.54 × 10^−103^**
F18	**5.62 × 10^−156^**	**4.49 × 10^−135^**	**1.94 × 10^−149^**	**1.88 × 10^−153^**	**2.56 × 10^−141^**	**8.67 × 10^−153^**	**2.49 × 10^−127^**	**4.88 × 10^−120^**
F19	**1.09 × 10^−160^**	**4.93 × 10^−155^**	**4.32 × 10^−146^**	**5.58 × 10^−156^**	**2.29 × 10^−157^**	**5.15 × 10^−159^**	**1.25 × 10^−153^**	**3.54 × 10^−110^**
F20	**7.01 × 10^−162^**	**3.83 × 10^−134^**	**2.52 × 10^−150^**	**5.67 × 10^−151^**	**1.71 × 10^−143^**	**6.24 × 10^−154^**	**3.98 × 10^−135^**	**4.67 × 10^−129^**
F21	**4.37 × 10^−157^**	**3.82 × 10^−146^**	**5.76 × 10^−149^**	**1.01 × 10^−154^**	**9.85 × 10^−155^**	**4.34 × 10^−156^**	**5.98 × 10^−155^**	**5.01 × 10^−148^**
F22	**9.04 × 10^−163^**	**1.01 × 10^−118^**	**9.31 × 10^−141^**	**1.46 × 10^−143^**	**2.18 × 10^−138^**	**1.01 × 10^−148^**	**1.65 × 10^−128^**	**1.36 × 10^−112^**
F23	**2.97 × 10^−161^**	**1.54 × 10^−129^**	**1.81 × 10^−151^**	**3.38 × 10^−152^**	**3.57 × 10^−147^**	**1.37 × 10^−152^**	**1.50 × 10^−134^**	**2.81 × 10^−130^**
F24	**4.54 × 10^−162^**	**2.78 × 10^−126^**	**2.35 × 10^−153^**	**3.11 × 10^−157^**	**6.62 × 10^−129^**	**4.21 × 10^−153^**	**3.39 × 10^−139^**	**5.74 × 10^−111^**
F25	**2.07 × 10^−164^**	**1.29 × 10^−148^**	**1.74 × 10^−162^**	**2.94 × 10^−156^**	**9.95 × 10^−132^**	**2.51 × 10^−128^**	**3.08 × 10^−79^**	**2.85 × 10^−135^**
F26	**8.37 × 10^−163^**	**9.21 × 10^−161^**	**1.26 × 10^−162^**	**1.43 × 10^−158^**	**1.89 × 10^−160^**	**1.50 × 10^−158^**	**3.79 × 10^−157^**	**3.40 × 10^−152^**
F27	**1.80 × 10^−161^**	**3.69 × 10^−135^**	**1.58 × 10^−153^**	**5.30 × 10^−156^**	**1.87 × 10^−141^**	**6.83 × 10^−152^**	**9.54 × 10^−143^**	**3.92 × 10^−127^**
F28	**5.30 × 10^−162^**	**2.97 × 10^−143^**	**1.32 × 10^−153^**	**1.59 × 10^−158^**	**2.82 × 10^−149^**	**2.71 × 10^−156^**	**1.98 × 10^−147^**	**6.82 × 10^−140^**
F29	**2.51 × 10^−159^**	**6.90 × 10^−141^**	**1.82 × 10^−152^**	**5.68 × 10^−156^**	**2.96 × 10^−146^**	**7.37 × 10^−155^**	**4.32 × 10^−155^**	**4.53 × 10^−136^**

**Table 8:** ImWOA’s sensitivity to the parameter changes.

Dim = 100				
Func.	Index	k = 0.4	k = 0.5	k = 0.6
F1	Min	1.06 × 10^9^	7.43 × 10^8^	7.90 × 10^8^
	Mean	3.98 × 10^10^	3.65 × 10^10^	3.80 × 10^10^
	Std	7.62 × 10^10^	7.61 × 10^10^	6.83 × 10^10^
	Rank	3	1	2
F2	Min	1.35 × 10^4^	1.41 × 10^4^	1.62 × 10^4^
	Mean	1.52 × 10^5^	1.30 × 10^5^	1.42 × 10^5^
	Std	1.87 × 10^5^	1.66 × 10^5^	1.77 × 10^5^
	Rank	3	1	2
F3	Min	1.15 × 10^3^	1.12 × 10^3^	1.33 × 10^3^
	Mean	1.07 × 10^4^	1.06 × 10^4^	1.18 × 10^4^
	Std	3.09 × 10^4^	2.28 × 10^4^	2.90 × 10^4^
	Rank	2	1	3
F4	Min	4.62 × 10^3^	4.11 × 10^3^	5.63 × 10^3^
	Mean	4.46 × 10^4^	4.08 × 10^4^	3.97 × 10^4^
	Std	8.46 × 10^4^	7.79 × 10^4^	7.30 × 10^4^
	Rank	3	2	1
F5	Min	5.00 × 10^2^	5.00 × 10^2^	5.00 × 10^2^
	Mean	5.00 × 10^2^	5.00 × 10^2^	5.00 × 10^2^
	Std	2.15 × 10^−2^	1.65 × 10^−2^	1.70 × 10^−2^
	Rank	3	2	1
F6	Min	4.14 × 10^4^	4.36 × 10^4^	3.19 × 10^4^
	Mean	1.55 × 10^6^	1.55 × 10^6^	1.55 × 10^6^
	Std	2.72 × 10^6^	2.85 × 10^6^	2.92 × 10^6^
	Rank	1	3	2
F7	Min	3.15 × 10^3^	3.00 × 10^3^	3.54 × 10^3^
	Mean	3.38 × 10^3^	3.30 × 10^3^	3.73 × 10^3^
	Std	2.57 × 10^2^	2.96 × 10^2^	2.33 × 10^2^
	Rank	2	1	3
F8	Min	9.51 × 10^2^	9.33 × 10^2^	9.67 × 10^2^
	Mean	1.03 × 10^3^	9.77 × 10^2^	1.03 × 10^3^
	Std	6.78 × 10^1^	4.61 × 10^1^	6.58 × 10^1^
	Rank	2	1	3
F9	Min	2.17 × 10^4^	2.12 × 10^4^	2.48 × 10^4^
	Mean	2.68 × 10^4^	2.76 × 10^4^	2.82 × 10^4^
	Std	2.19 × 10^3^	2.71 × 10^3^	2.19 × 10^3^
	Rank	1	2	3
F10	Min	3.71 × 10^5^	3.02 × 10^5^	3.83 × 10^5^
	Mean	4.64 × 10^9^	3.62 × 10^9^	4.34 × 10^9^
	Std	2.05 × 10^10^	1.97 × 10^10^	2.50 × 10^10^
	Rank	3	1	2
F11	Min	1.48 × 10^9^	8.86 × 10^8^	7.18 × 10^8^
	Mean	1.34 × 10^10^	1.23 × 10^10^	9.76 × 10^9^
	Std	3.34 × 10^10^	3.43 × 10^10^	3.33 × 10^10^
	Rank	3	2	1
F12	Min	1.04 × 10^8^	1.12 × 10^8^	2.01 × 10^8^
	Mean	2.75 × 10^10^	2.54 × 10^10^	2.44 × 10^10^
	Std	9.50 × 10^10^	9.29 × 10^10^	8.25 × 10^10^
	Rank	3	2	1
F13	Min	5.18 × 10^6^	3.82 × 10^6^	5.43 × 10^6^
	Mean	3.16 × 10^7^	2.34 × 10^7^	1.47 × 10^7^
	Std	7.04 × 10^7^	8.05 × 10^7^	3.01 × 10^7^
	Rank	3	2	1
F14	Min	2.09 × 10^7^	1.62 × 10^7^	2.75 × 10^7^
	Mean	3.99 × 10^9^	2.52 × 10^9^	2.90 × 10^9^
	Std	1.56 × 10^10^	1.31 × 10^10^	1.18 × 10^10^
	Rank	3	1	2
F15	Min	9.82 × 10^4^	7.97 × 10^4^	1.14 × 10^5^
	Mean	1.29 × 10^9^	5.34 × 10^8^	3.85 × 10^8^
	Std	6.38 × 10^9^	3.87 × 10^9^	2.33 × 10^9^
	Rank	3	2	1
F16	Min	1.52 × 10^5^	3.02 × 10^5^	1.46 × 10^5^
	Mean	1.82 × 10^14^	9.87 × 10^14^	3.26 × 10^14^
	Std	1.92 × 10^15^	1.10 × 10^16^	2.96 × 10^15^
	Rank	1	3	2
F17	Min	1.90 × 10^7^	1.09 × 10^7^	1.27 × 10^7^
	Mean	8.65 × 10^7^	8.76 × 10^7^	1.06 × 10^8^
	Std	2.96 × 10^8^	3.12 × 10^8^	3.99 × 10^8^
	Rank	1	2	3
F18	Min	2.15 × 10^6^	1.95 × 10^6^	2.35 × 10^6^
	Mean	7.77 × 10^14^	3.09 × 10^14^	3.01 × 10^14^
	Std	7.33 × 10^15^	4.73 × 10^15^	2.98 × 10^15^
	Rank	3	2	1
F19	Min	1.12 × 10^4^	9.23 × 10^3^	1.63 × 10^4^
	Mean	1.35 × 10^4^	1.20 × 10^4^	1.78 × 10^4^
	Std	4.65 × 10^3^	5.19 × 10^3^	2.85 × 10^3^
	Rank	2	1	3
F20	Min	7.04 × 10^3^	7.15 × 10^3^	8.22 × 10^3^
	Mean	3.75 × 10^4^	3.49 × 10^4^	4.02 × 10^4^
	Std	5.57 × 10^4^	6.10 × 10^4^	7.00 × 10^4^
	Rank	2	1	3
F21	Min	1.80 × 10^4^	1.89 × 10^4^	1.48 × 10^4^
	Mean	2.28 × 10^4^	2.34 × 10^4^	1.84 × 10^4^
	Std	3.28 × 10^3^	2.34 × 10^3^	3.23 × 10^3^
	Rank	2	3	1
F22	Min	6.30 × 10^3^	5.47 × 10^3^	5.97 × 10^3^
	Mean	2.91 × 10^4^	2.72 × 10^4^	3.21 × 10^4^
	Std	4.15 × 10^4^	3.76 × 10^4^	4.21 × 10^4^
	Rank	2	1	3
F23	Min	7.24 × 10^3^	7.72 × 10^3^	7.85 × 10^3^
	Mean	3.94 × 10^4^	4.60 × 10^4^	4.77 × 10^4^
	Std	5.29 × 10^4^	6.02 × 10^4^	5.65 × 10^4^
	Rank	1	2	3
F24	Min	3.93 × 10^3^	4.27 × 10^3^	4.25 × 10^3^
	Mean	6.77 × 10^3^	7.33 × 10^3^	7.45 × 10^3^
	Std	8.52 × 10^3^	8.77 × 10^3^	9.07 × 10^3^
	Rank	1	2	3
F25	Min	8.89 × 10^3^	1.20 × 10^4^	6.24 × 10^3^
	Mean	1.96 × 10^4^	2.61 × 10^4^	1.93 × 10^4^
	Std	3.54 × 10^4^	4.12 × 10^4^	3.91 × 10^4^
	Rank	2	3	1
F26	Min	7.05 × 10^3^	4.40 × 10^3^	4.31 × 10^3^
	Mean	7.56 × 10^3^	4.99 × 10^3^	4.87 × 10^3^
	Std	6.10 × 10^2^	1.00 × 10^3^	9.48 × 10^2^
	Rank	3	2	1
F27	Min	3.31 × 10^3^	3.33 × 10^3^	3.29 × 10^3^
	Mean	5.65 × 10^3^	5.57 × 10^3^	5.47 × 10^3^
	Std	5.53 × 10^3^	5.34 × 10^3^	5.99 × 10^3^
	Rank	3	2	1
F28	Min	1.93 × 10^8^	8.22 × 10^7^	2.33 × 10^8^
	Mean	2.24 × 10^14^	3.06 × 10^14^	6.95 × 10^14^
	Std	2.62 × 10^15^	3.69 × 10^15^	6.87 × 10^15^
	Rank	1	2	3
F29	Min	1.84 × 10^8^	2.76 × 10^8^	2.32 × 10^8^
	Mean	2.92 × 10^15^	1.42 × 10^14^	5.01 × 10^14^
	Std	2.85 × 10^16^	1.61 × 10^15^	5.91 × 10^15^
	Rank	3	1	2
Average Rank		2.241	1.759	2
Combined Rank		3	1	2

**Table 9:** Results for the reducer design problem. Bold text indicates the optimal values.

Algorithm	ImWOA	CSA	HHO	EHO	BES	WOA	ImWOA1	ImWOA2	ImWOA3
Min	2989.849257	3187.504612	3017.579357	3233.653857	3005.3245	2988.375969	3216.124998	3003.738979	3117.319136
Mean	**2997.190731**	3287.342834	3066.271203	3323.970413	3007.73153	3013.680497	3241.422345	3017.044984	3122.004995
Std	65.4827813	137.1478562	132.6657384	117.8959109	23.15272402	100.0657849	132.0832982	29.44948724	6.91776239
Rank	1	8	5	9	2	3	7	4	6

**Table 10:** Results for vehicle side impact design problem. Bold text indicates the optimal values.

Algorithm	ImWOA	CSA	HHO	EHO	BES	WOA	ImWOA1	ImWOA2	ImWOA3
Min	23.44509133	27.69061235	28.77157096	27.30036156	26.47086361	29.20482798	28.64094369	24.79899961	28.92041904
Mean	**24.15181044**	40.36471146	29.44543138	28.05705143	26.57339049	30.07469847	29.17769936	26.29671769	29.11228986
Std	0.669615869	108.7740011	1.57316929	2.659410249	0.796139692	1.457683074	2.575157747	1.97214199	0.419476751
Rank	1	9	7	4	3	8	6	2	5

**Table 11:** Results for welded beam design problem. Bold text indicates the optimal values.

Algorithm	ImWOA	CSA	HHO	EHO	BES	WOA	ImWOA1	ImWOA2	ImWOA3
Min	1.793862002	2.418196655	2.987921052	2.359222519	1.967963538	3.252537478	2.250407104	1.743418076	2.902846975
Mean	**1.957473208**	40.11746797	98.0714428	4.217360109	12.46814113	19.63870544	35.08390682	8.114092921	2.910706402
Std	0.241309368	373.6671073	1063.002607	28.90688132	226.044931	84.71099269	251.9895056	104.5104734	0.044455212
Rank	1	8	9	3	5	6	7	4	2

## Data Availability

The raw data supporting the conclusions of this article will be made available by the authors on request.
